# *Eleutherococcus* root: a comprehensive review of its phytochemistry and pharmacological potential in the context of its adaptogenic effect

**DOI:** 10.3389/fphar.2025.1683795

**Published:** 2025-10-29

**Authors:** Andrzej Patyra, Małgorzata Kołtun-Jasion, Katarzyna Kupniewska, Andrzej Parzonko, Anna Karolina Kiss

**Affiliations:** Department of Pharmaceutical Biology, Faculty of Pharmacy, Medical University of Warsaw, Warsaw, Poland

**Keywords:** *Eleutherococcus senticosus*, eleuthero, Araliaceae, eleutherosides, quality control, adaptogen

## Abstract

*Eleutherococcus senticosus* (Araliaceae) has been known as a traditional medicine for twenty centuries. Various preparations of *E. senticosus* root are available as an adaptogen to reduce fatigue and stress, decrease blood glucose levels, and stimulate the immune system. The European Medicines Agency approved *E. senticosus* root for the treatment of symptoms of asthenia, such as fatigue and weakness. This review compiles the phytochemistry of the root, as well as the quality assessment of plant material and commercial products with recent reports on biological activities, in terms of relief symptoms of asthenia and immunomodulating effect. Moreover, the clinical evidence of *E. senticosus* preparations for the treatment of symptoms of asthenia as an adaptogen was critically reviewed. The pharmacological effect of *E. senticosus* is connected with various constituents, among prevail caffeoylquinic acids, a phenylpropanoid-syringin (eleutheroside B), and syringaresinol derivatives. Two marker compounds, eleutheroside B and eleutheroside E (syringaresinol diglucoside), are used to standardize the eleuterococcus products. Especially, syringin appears as a specific marker for discrimination from other Araliaceae plant materials. The preclinical studies demonstrated that the adaptogenic action of *Eleutherococcus* root is a combination of anti-inflammatory and immunomodulatory effects (inhibition of MAPKs, Akt, and NF-κB activation), and neuroprotective activity (increase of brain-derived neurotrophic factor), which may contribute to the stress/fatigue reduction and memory-enhancing effects. Eleutherosides B and E appear to be of particular importance in modulating the adaptogenic response. The main issue is the lack of robust clinical evidence for the treatment of symptoms of asthenia as an adaptogen. The high heterogeneity and low quality of clinical trials in connection with the lack of proper standardization of *E. senticosus* preparations make impossible to assess the effectiveness. Moreover, the poor quality of preparations may strongly influence the efficacity of this interesting medicinal plant.

## 1 Introduction

*Eleutherococcus senticosus* (Rupr. and Maxim.) Maxim (*Acanthopanax senticosus* (Rupr. and Maxim.) Harms), also called Siberian ginseng, Shigoka, or Ciwujia, has been known as a traditional medicine for twenty centuries. The alternative name ‘Siberian ginseng’ was introduced in the USA and refers to the fact that *E. senticosus* was exported for the first time from Siberia in the Soviet era. However, this term was not used by the local population, as they call this plant eleutherokokk (Davydov and Krikorian, 2000). Some authors claim that the designation Siberian ginseng is misleading because *E. senticosus* is only distantly related to *Panax ginseng* C.A.Mey (Asian/Chinese/Japanese/Korean ginseng) and *Panax quinquefolius* L. (American ginseng) and differs from them in terms of chemical composition (Davydov and Krikorian, 2000; Huang et al., 2011a). Moreover, the formally accepted common name for this species in the US is “eleuthero”, and it is illegal to refer to eleuthero products as “ginseng”.

This medicinal plant is widely distributed in Asia, especially in Northeast China, North Japan, and Korea, Southeast Russia, and the Russian Far East (Huang et al., 2021). *Eleutherococcus senticosus* belongs to the Araliaceae family. *Eleutherococcus* is a large genus that includes almost 40 different species (Online TWF). The Latin name of the plant adequately describes it. *Eleutherococcus* is derived from Greek, *eleutheros,* which means free, *kokkos* meaning pip, seed, or a pyrene according to botanical terminology, and Latin *senticosus* meaning “full of briers or thorns”. Interestingly, the former Latin name *A. senticosus* was also connected with this anatomical feature: *Acanthopanax* = thorny ginseng. *Eleutherococcus senticosus* is a shrub that may grow between 2 and 7 m tall, usually with several mostly unbranched stems (Davydov and Krikorian, 2000). Its branches are armed with about 5 mm dense or scattered prickles. *Eleutherococcus senticosus* has compound leaves comprising three to five leaflets with thorned petioles, and each leaflet is elliptic-obovate, or oblong in shape. Its inflorescence is terminal, a solitary or compound umbel. The corolla is purple-yellow in color (Huang et al., 2011a). Fruit, a black drupe (diameter about 10 mm), contains an equal number of kernels and carpels. Flowers and fruits are similar to those of ivy (*Hedera helix* L. - Araliaceae) (WHO, 2002; Baranov, 1982). The leaf of *E. senticosus* has been shown to have medicinal properties. However, its root is the most frequently used part of the plant (Murray, 2020). The highest content of active constituents in the root is detected in the autumn, just before defoliation (Baranov, 1982). The root plant material is of irregular cylindrical shape, up to 0.5 cm in diameter, grayish brown to dark brown, smooth surface with bark adhering closely to the xylem, Various preparations of *E. senticosus* root are available: tincture, liquid and dry hydro-alcoholic extracts (capsules, tablets), teas, and powders. Producers recommend a daily dose ranging from 0.5 g to 1.5 g of plant material. According to the EMA monograph, a daily dose should be in the range of 0.5–4 g of dried root (European Medicines Agency, 2014). Manufacturers indicate that the root of *E. senticosus* effectively reduces fatigue and stress, decreases blood glucose levels, and stimulates the immune system. There are also several combinations of *E. senticosus* root with *Panax quinquefolius* root, *Withania somnifera* (L.) Dunal root (Ashwagandha) and *Panax ginseng* root.

**FIGURE 1:**
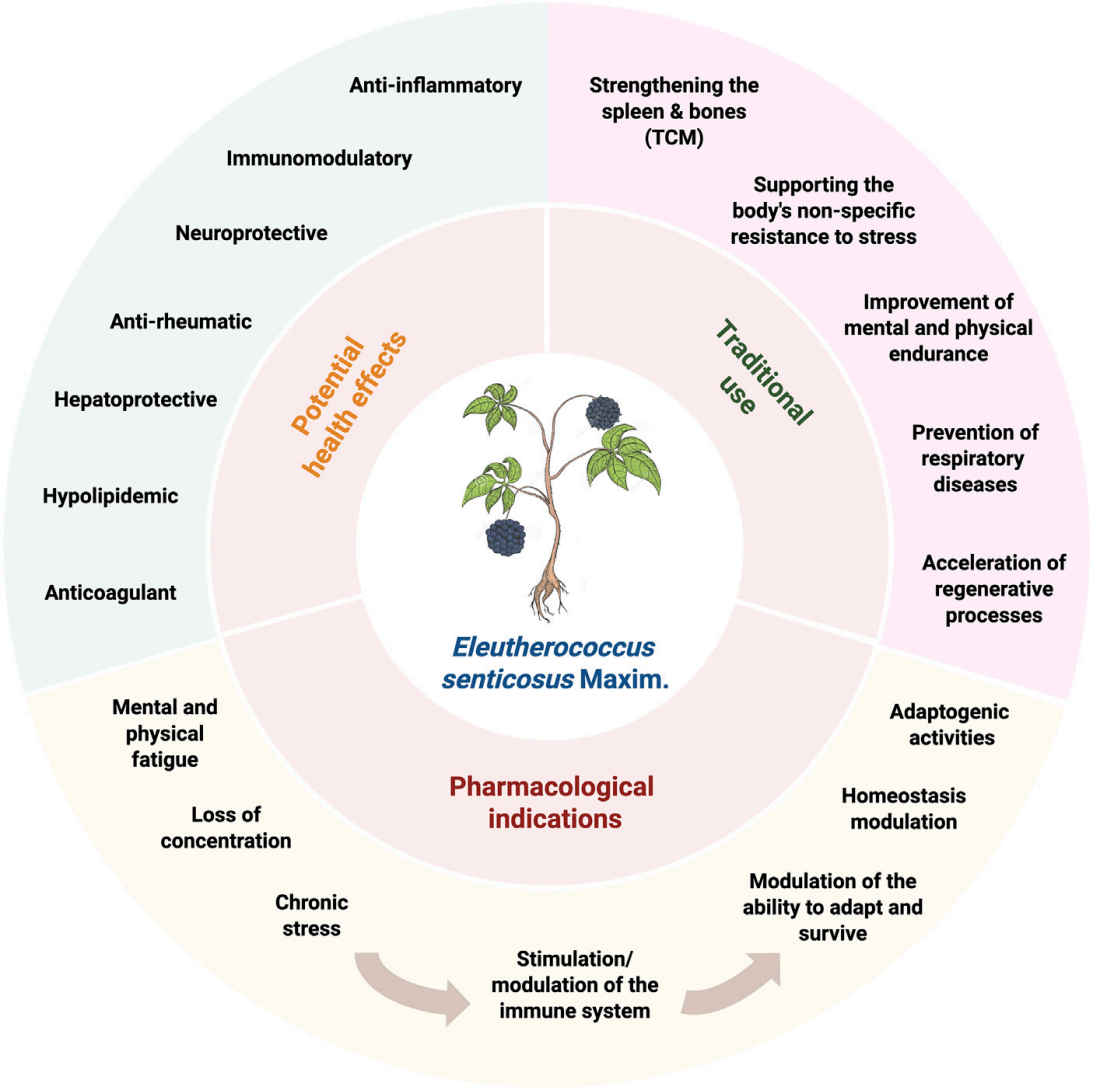
Role of *E. senticosus* in phytomedicine.

*Eleutherococcus senticosus*, is an important medicinal plants, which was reported to have several potential health effects (Figure 1). Although, there have been some reviews concerning *E. senticosus* phytochemistry and pharmacology (Davydov and Krikorian, 2000; Huang et al., 2011a; Jia et al., 2021; Bleakney, 2008; Li et al., 2016; Yan-Lin et al., 2011), none of them concentrated on the root of this plant, which is the only part approved by The European Medicines Agency (EMA) and being mentioned in Western pharmacopeias. This review compiles the ethnopharmacological uses of this plant, chemical constituents isolated from the root of *E. senticosus,* as well as the quality assessment of plant material and commercial products. Recent reports on the biological activities of its extracts (Figure 1) and major active constituents, in terms of relief of symptoms of asthenia and immunomodulating effect, are also discussed (Figure 1). Moreover, the clinical evidence of *E. senticosus* preparations for the treatment of symptoms of asthenia as an adaptogen was critically reviewed.

## 2 Ethnopharmacology

The first description of *E. senticosus* appeared in ‘Shennong Bencao Jing’, a Chinese book dated back to 100 BC – 200 AD or even to 2000 BC (Huang et al., 2021; Baranov, 1982). Later*, E. senticosus* was included in many monographs and is listed now in the Chinese Pharmacopoeia (Huang et al., 2021). From the perspective of traditional Chinese medicine (TCM), *E. senticosus* is used to nourish qi, which is a vital source of energy for the human organism (Jia et al., 2021; Li et al., 2013). In TCM theory*, E. senticosus* fortifies the spleen, strengthens the bones, and calms the mind (Jia et al., 2021).

The story of a broad-scale use of *E. senticosus* in Europe began in Russia. At first, a physician, P.E. Kirilov, brought *E. senticosus* from Beijing in 1842. Later, the plant was botanically described by C.I. Maximowicz. In the late 1940s, Russian/Soviet scientists searched for compounds that give the organism a ‘non-specifically increased resistance’ in experimental animals and humans (Shikov et al., 2021). The term adaptogen was used for the first time by the Russian toxicologist N. Lazarev in 1958. It was reserved for substances capable of stimulating the resilience of organisms to stress (Panossian et al., 2021; Lazarev, 1958). Brekhman and Dardymov in 1969 listed such adaptogens as *E. senticosus*, *Panax ginseng*, *Rhodiola rosea* L., *Schisandra chinensis* (Turcz.) Baill., and others. According to these researchers, there are three features of adaptogens: 1) they cannot be harmful, and they should have a minimal impact on the physiological functions of an organism; 2) their action should be nonspecific–they should enhance resistance to unfavorable influences of many factors of physical, chemical, and biological nature; 3) they may exert a normalizing effect regardless of the direction of previous changes. The *E. senticosus* root was found to comply with the requirements listed above (Brekhman and Dardymov, 1969).

As wild *P. ginseng* was rarely found in Eastern Siberia, Korea, and adjacent areas, Russian/Soviet scientists aimed at identifying alternative plant material with similar biological activity, and *E. senticosus* was considered as such a substitute (Davydov and Krikorian, 2000). Thus, many studies of its adaptogenic properties have been conducted in the former Soviet Union (nowadays Russia). *Eleutherococcus senticosus* was listed for the first time in the pharmacopeia of the Soviet Union in 1962. A recent review compiled forty-six studies published between 1962 and 1986 in the Soviet Union that previously were not translated into English (Gerontakos et al., 2021). Although the described research work has many limitations, *E. senticosus* was shown to improve cognitive function and physical and mental endurance and prevent respiratory diseases. These findings also suggest that *E. senticosus* is a safe and well-tolerated herbal medicine. Since then, many more studies on this plant have been carried out.

In 2002, the World Health Organization (WHO) *monographs on selected medicinal plants* were published, presenting that *E. senticosus* root is effective (according to clinical data) in reducing exhaustion and tiredness and can speed up the recovery process (WHO, 2002). The European Medicines Agency (EMA) approved *E. senticosus* root in 2014 for the treatment of symptoms of asthenia, such as fatigue and weakness. It was classified as a traditional herbal medicine entirely based on its long-standing use (Community herbal monograph on *Eleutherrococcus*). The 14th edition of the State Pharmacopoeia of the Russian Federation contains a monograph for *E. senticosus* (root and rhizome), which is marked there as a tonic and an adaptogen (State Pharmacopoeia of Russian Federation, 2018). The root of *E. senticosus* has been listed in the 10th edition of the European Pharmacopeia (European Pharmacopoeia, 2019), the European Scientific Cooperativeon Phytotherapy Monographs (ESCOP, 2003), and in the British Pharmacopoeia (British Pharmacopoeia Commission, 2022). The monograph for *E. senticosus* rhizome is included in the Japanese Pharmacopoeia 18th edition (Japanese Pharmacopoeia, 2021).

## 3 Phytochemistry

Instead of having one dominant compound or group of metabolites, *E. senticosus* root has a mixture of different constituents, of which the major ones are sometimes referred to as eleutherosides. Among these eleutherosides are carbohydrates or glycosides of phenolic alcohols, lignans, coumarins, and triterpenoids. However, they are not limited to this plant and can be found in many different species, sometimes in higher quantities than in eleuthero. There is even some debate about whether they can be considered as the main active constituents, as many studies have shown that the root is far richer in hydroxycinnamic acid derivatives than in any of the eleutherosides. Basically, there are eleutherosides A-M. Eleutherosides are present in various parts of *E. senticosus*, from root to stem (Abbai et al., 2016; Deyama et al., 2001), fruit pulp (Yan et al., 2014), leaf (Deyama et al., 2001; Jin et al., 2004), rhizome (Bai et al., 2011), and bark (Jin et al., 2020). These compounds can be classified into two groups (Davydov and Krikorian, 2000). The first one includes phenylpropanoid derivatives such as eleutherosides B (sinapyl alcohol 4-*O*-β-D-glucoside: syringin), B1 (isofraxidin-7-*O*-β-D-glucoside), B4 (sezamin), D, E (syringaresinol di-*O*-β-D-glucoside), E1 (syringaresinol-*O*-β-D-glucoside, and E2 (episyringaresinol-*O*-β-D-glucoside). The second group consists of eleutherosides I-M, which are the triterpene saponins (glycosides of oleanolic acid) (Davydov and Krikorian, 2000). Eleutheroside D (syn. acanthoside D, liriodendrin, syringaresinol di-*O*-β-D-glucoside) and eleutheroside E are diastereomers that have different configurations at C-7 and C-8 (WHO, 2002; Kil et al., 2015). Although eleutherosides F and G are listed as *E. senticosus* constituents, there were no reports about their structure (Feng et al., 2006). To conclude, most of the eleutherosides are important only from a historical perspective, except for eleutherosides B and E, which are marker compounds for *E. senticosus* and are considered to be responsible for its antifatigue and other effects (Huang L. Z. et al., 2011).

As previously described, eleutherosides B and E are used for the standardization of this plant material. As mentioned in the European Pharmacopoeia monograph of *E. senticosus*, its dried whole or cut underground organs should contain a minimum of 0.08% for the sum of eleutheroside B and eleutheroside E (European Pharmacopoeia, 2019).

**FIGURE 2:**
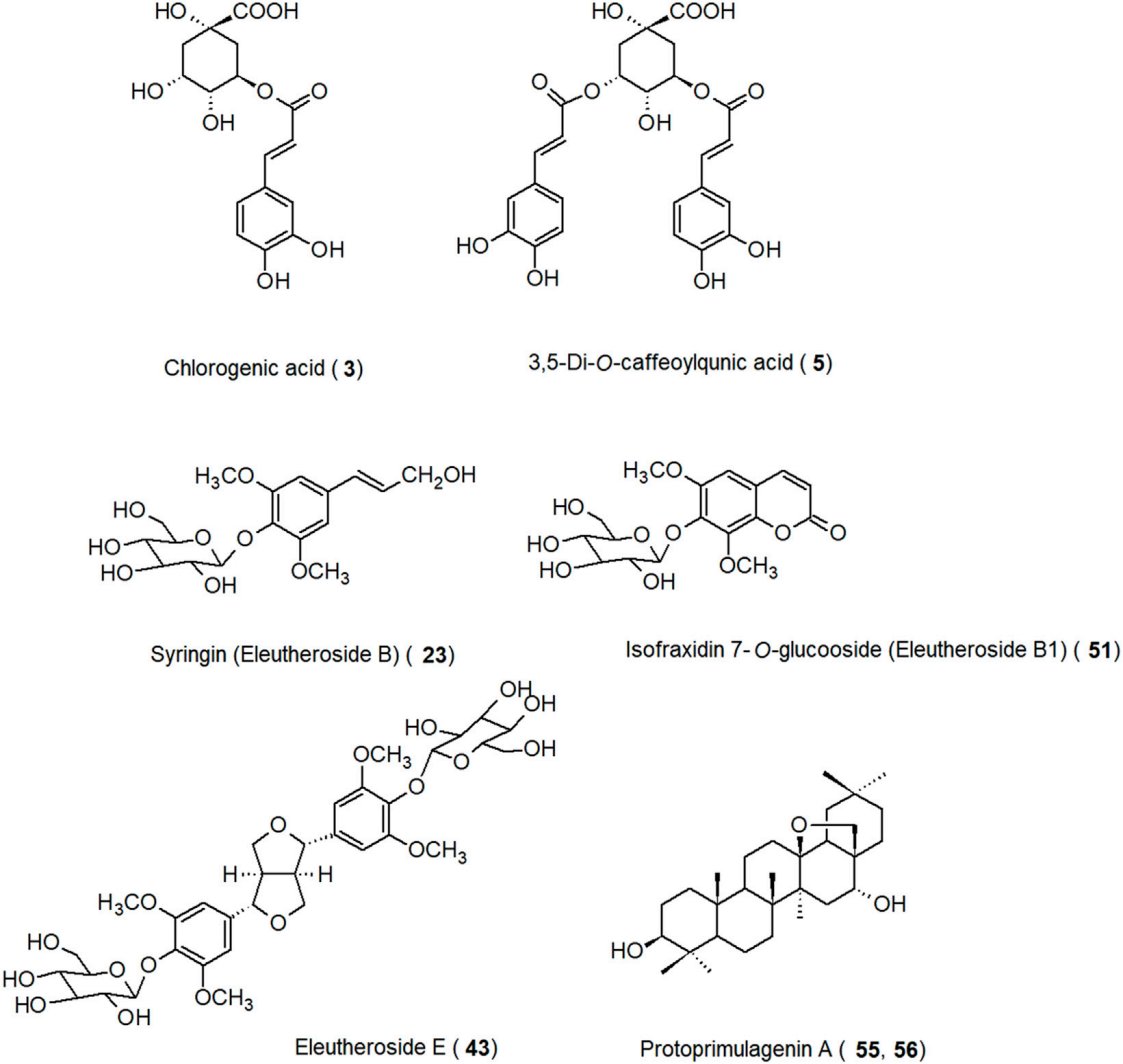
Selected major constituents isolated from *E. senticosus* root.

Fifty-eight compounds have been identified from *E. senticosus* root. Their chemical names and classes are listed in Table 1. Selected major constituents isolated from *E. senticosus* root are presented in Figure 2. The root of *E. senticosus* contains phenolic acids and their derivatives, lignans as major components, as well as coumarins and triterpenoid saponins in lower quantities.

**TABLE 1:** Secondary metabolites identified in the root of *Eleutherococcus senticosus*.

Classification	No.	Chemical component	References
Phenolic acids and their derivatives	1.	1-*O*-Caffeoylquinic acid	Shi et al. (2020)
	2.	1,5-Di-*O*-caffeoylquinic acid (Cynarin; 1,3-di-*O*-caffeoylquinic acid)	Shi et al. (2020), Slacanin et al. (1991), Tolonen et al. (2002)
	3.	3-*O*-Caffeoylquinic acid (Chlorogenic acid)	Deyama et al. (2001), Shi et al. (2020), Slacanin et al. (1991), Kurkin et al. (1991), He et al. (2020), Liu et al. (2018), Zgórka and Kawka (2001), Kim et al. (2015)
	4.	3,4-Di-*O*-caffeoylquinic acid (Isochlorogenic acid B)	Shi et al. (2020)
	5.	3,5-Di-*O*-caffeoylquinic acid (Isochlorogenic acid A)	Shi et al. (2020), Tolonen et al. (2002)
	6.	4,5-Di-*O*-caffeoylquinic acid (Isochlorogenic acid C)	Tolonen et al. (2002)
	7.	5-*O*-Caffeoylquinic acid (Neochlorogenic acid)	Tolonen et al. (2002), Liu et al. (2018)
	8.	Caffeic acid	Kurkin et al. (1991), Zgórka and Kawka (2001), Kim et al. (2015), Su et al. (2021)
	9.	Coniferaldehyde	Slacanin et al. (1991), Su et al. (2021)
	10.	Coniferaldehyde 4-*O*-β-D-glucoside	Slacanin et al. (1991)
	11.	Coniferin	Slacanin et al. (1991), Kurkin et al. (1991)
	12.	Coniferyl alcohol	Kurkin et al. (1991)
	13.	Cryptochlorogenic acid (4-*O*-Caffeoylquinic acid)	Shi et al. (2020)
	14.	Ferulic acid	Shi et al. (2020), Kurkin et al. (1991), Zgórka and Kawka (2001)
	15.	Gentisic acid	Su et al. (2021)
	16.	*p*-Coumaric acid	Kurkin et al. (1991), Zgórka and Kawka (2001), Su et al. (2021)
	17.	*p*-Hydroxybenzoic acid	Kurkin et al. (1991), Zgórka and Kawka (2001)
	18.	Protocatechuic acid	Zgórka and Kawka (2001)
	19.	Quinic acid	He et al. (2020)
	20.	Sinapaldehyde 4-*O*-glucoside	Slacanin et al. (1991)
	21.	Sinapyl alcohol	Slacanin et al. (1991)
	22.	Syringic acid	Kurkin et al. (1991), Zgórka and Kawka (2001)
	23.	Syringin (Eleutheroside B, Sinapyl alcohol 4-*O*-β-D-glucoside)	Deyama et al. (2001), Shi et al. (2020), Slacanin et al. (1991), Kurkin et al. (1991), Liu et al. (2018), Li et al. (2001)
	24.	trans-Cinnamaldehyde	Shi et al. (2020)
	25.	Vanillic acid	Kurkin et al. (1991), Zgórka and Kawka (2001)
	26.	Vanillin	Shi et al. (2020), Kurkin et al. (1991), Su et al. (2021)
Lignans	27.	7,8-dihydrodehydrodiconiferyl alcohol	Makarieva et al. (1997)
	28.	7,8-trans-dihydrodehydrodiconiferyl alcohol 4-*O*-β-D-glucoside	Makarieva et al. (1997)
	29.	Asarinin	Shi et al. (2020)
	30.	Dehydrodiconiferyl alcohol	Makarieva et al. (1997)
	31.	Eleutheroside E2 (Episyringaresinol 4′-*O*-β-D-glucoside)	Huang et al. (2011b), Li et al. (2001)
	32.	Matairesinol	Su et al. (2021)
	33.	Medioresinol 4,4′-di-*O*-β-D-glucoside	Deyama et al. (2001)
	34.	Medioresinol 4′-*O*-β-D-glucoside	He et al. (2020)
	35.	Pinoresinol	Shi et al. (2020)
	36.	Pinoresinol 4-*O*-β-D-glucoside	Deyama et al. (2001)
	37.	Pinoresinol 4,4′-di-*O*-β-D-glucoside	Deyama et al. (2001), He et al. (2020)
	38.	Secoisolariciresinol	Makarieva et al. (1997)
	39.	Sesamin (Eleutheroside B4)	Jin et al. (2020), Shi et al. (2020), Slacanin et al. (1991)
	40.	Syringaresinol (Lirioresinol B)	Slacanin et al. (1991), Kurkin et al. (1991), Su et al. (2021)
	41.	Syringaresinol 4-*O*-β-D-apiosyl-(1→2)-β-D-glucoside	Shi et al. (2020)
	42.	Syringaresinol 4-*O*-β-D-glucoside (Eleutheroside E1, Acanthoside B)	Deyama et al. (2001), He et al. (2020), Su et al. (2021), Li et al. (2001), Makarieva et al. (1997)
	43.	Syringaresinol 4,4′-di-*O*-β-D-glucoside (Eleutheroside E, Eleutheroside D, Liriodendrin)	Deyama et al. (2001), Huang et al. (2011b), Shi et al. (2020), Slacanin et al. (1991), Kurkin et al. (1991), He et al. (2020), Liu et al. (2018), Su et al. (2021), Li et al. (2001)
Coumarins	44.	6,7-dihydroxy-4-methylcoumarin (4-Methylesculetin)	Shi et al. (2020)
	45.	6,7,8-Trimethoxycoumarin (Dimethylfraxetin)	Su et al. (2021)
	46.	Esculin	Shi et al. (2020)
	47.	Fraxetin	Shi et al. (2020)
	48.	Fraxidin	Su et al. (2021)
	49.	Fraxinol	Shi et al. (2020), Su et al. (2021)
	50.	Isofraxidin	Deyama et al. (2001), Shi et al. (2020), Slacanin et al. (1991), Kurkin et al. (1991), He et al. (2020), Liu et al. (2018), Su et al. (2021)
	51.	Isofraxidin 7-*O*-β-D-glucoside (Eleutheroside B1)	Deyama et al. (2001), Slacanin et al. (1991), Kurkin et al. (1991), Liu et al. (2018)
	52.	Scoparone (6,7-Dimethoxycoumarin)	Shi et al. (2020)
	53.	Umbelliferone	Makarieva et al. (1997)
Other compounds	54.	Phlorizin (Phloridzin)	Lee et al. (2013), Choi et al. (2016)
	55.	Protoprimulagenin A 3β-[*O*-α-L-rhamnosyl-(1→4)-*O*-α-L-rhamnosyl-(1→4)-[*O*-α-L-rhamnosyl-(1→2)]-*O*-β-D-glucosyl-(1→x)-*O*-β-D-glucuronide]	Segiet-Kujawa and Kaloga (1991)
	56.	Protoprimulagenin A 3β-[*O*-β-D-glucosyl-(1→3)-*O*-β-D-galactosyl-(1→4)-[*O*-α-L-rhamnosyl-(1→2)]-*O*-β-D-glucuronide]	Segiet-Kujawa and Kaloga (1991)
	57.	Eleutheroside A (Daucosterol, β-Sitosterol glucoside, Sitogluside)	Slacanin et al. (1991)
	58.	β-sitosterol	Slacanin et al. (1991)

### 3.1 Phenolic acids and their derivatives

Phenolic acids, their derivatives, and glucosides are the major constituents of the *E. senticosus* and were among the first to be isolated from this plant material. In a study by Kim *et al.*, total phenolic content expressed as milligrams of gallic acid equivalents (GAE) per gram of sample in the case of the root extract was 44.00 mg GAE/dry weight g (Kim et al., 2015). Chlorogenic acid is one of the compounds with the highest content, ranging from 0.1% to 0.5% (Deyama et al., 2001; Slacanin et al., 1991; Zgórka and Kawka, 2001; Kim et al., 2015). Syringin (eleutheroside B) is one of two marker compounds for *E. senticosus* in combination with eleutheroside E; its content in the root varied from 0.01% to 0.04% (Deyama et al., 2001; Slacanin et al., 1991). Quantitative analysis performed using high-performance liquid chromatography (HPLC) showed that in the root, the content of coniferin, sinapaldehyde 4-*O*-glucoside, coniferaldehyde, and cynarin was 0.008%, 0.007%, 0.006%, and 0.003%, respectively (Slacanin et al., 1991).

### 3.2 Lignans

Lignans identified in *E. senticosus* root mainly included coniferyl alcohol derivatives and glycosides of medioresinol, pinoresinol, and syringaresinol. Eleutheroside E (syringaresinol 4,4′-di-*O*-β-D-glucoside) which is used for the standardization of *E. senticosus* products, was present in the root at concentrations ranging from 0.006% to 0.01% (Deyama et al., 2001; Huang L. Z. et al., 2011; Slacanin et al., 1991). Eleutheroside E2 (episyringaresinol 4′-*O*-β-D-glucoside) was isolated for the first time from *E. senticosus* root (Li et al., 2001), and its content there was 0.001% (Huang L. Z. et al., 2011).

### 3.3 Coumarins

Coumarins form another group of secondary metabolites that have been isolated from the root of *E. senticosus*. Isofraxidin is a hydroxy coumarin that has been isolated for the first time from the bark of the ash tree (*Fraxinus excelsior* L., Oleaceae) and later from many plant families such as Apiaceae, Asteraceae, Oleaceae, and Rubiaceae (Majnooni et al., 2020). Its content in the root of *E. senticosus* was 0.006% (Deyama et al., 2001), while the content of isofraxidin 7-*O*-β-D-glucoside (eleutheroside B1) was in the range of 0.012%–0.02% (Deyama et al., 2001; Slacanin et al., 1991).

### 3.4 Minor constituents

Root of *E. senticosus* also contains flavonoids, triterpenoid saponins, and steroids.

As Kim *et al.* described, the flavonoid content of the root extract expressed as milligrams of rutin equivalents (RE) per gram of sample was 36.49 mg RE/dry weight g (Kim et al., 2015). From flavonoids isolated from *E. senticosus* root, phloridzin content was found to be about 0.013% (Lee et al., 2013; Choi et al., 2016).

Triterpenoid saponins were generally found in the leaf and fruit of *E. senticosus* (Liu et al., 2020; Jiang et al., 2006; Xia et al., 2019; Li et al., 2007)*.* However, there is one report on the identification of two protoprimulagenin A glycosides in the root. Their content ranged between 0.0011% and 0.009% (Segiet-Kujawa and Kaloga, 1991).

### 3.5 Primary metabolites–polysaccharides

Seven glycans named eleutherans A-G were isolated from the root of *E. senticosus* (Hikino et al., 1986). The monosaccharide composition of these compounds was determined and is presented in Table 2. A small content of *O*-acetyl groups was reported in eleutherans A, B, C, E, F, and G (1.1, 2.2, 1.1, 1.7, 4.2, and 1.9%, respectively). The Lowry method was used for the determination of peptide moieties in eleutherans A-G (2.7, 3.2, 5.4, 5.9, 0.9, 6.2, and 5.8, respectively).

**TABLE 2:** Structural characterization of polysaccharides identified from the root of *E. senticosus*.

No.	Compound name	Monosaccharide composition (with molar ratio)	References
1	eleutheran A	Rha:Ara:Xyl:Man:Gal:Glc (0.3:0.1:0.2:3.6:1.0:1.4)	Hikino et al. (1986)
2	eleutheran B	Rha:Ara:Man:Gal:Glc (0.6:0.1:0.1:1.0:0.8)	Hikino et al. (1986)
3	eleutheran C	Rha:Ara:Man:Gal:Glc (0.9:0.6:0.3:1.0:2.7)	Hikino et al. (1986)
4	eleutheran D	Rha:Ara:Man:Gal:Glc (0.1:0.5:0.2:1.0:0.3)	Hikino et al. (1986)
5	eleutheran E	Rha:Ara:Xyl:Gal:Glc:GalA:GlcA (2.0:1.5:0.1:1.0:0.1:2.2:1.0)	Hikino et al. (1986)
6	eleutheran F	Ara:Man:Gal:Glc (0.6:0.2:1.0:0.7)	Hikino et al. (1986)
7	eleutheran G	Rha:Ara:Man:Gal:Glc (0.3:0.1:4.0:1.0:0.9)	Hikino et al. (1986)
8	CASPs	Ara:Man:Rha:Gal:Glu (1:1.1:3:4.7:5.5)	Xie et al. (2015)
9	PES-A	Glc:Gal:Ara (3.3:2:1)	Li et al. (2021)
10	As-III	Ara:Xyl:4-O-methyl-D-GlcA (1:11:1)	Li et al. (2021)
11	AS-2	Ara:Xyl:Rha:Gal:Glc (1.6:1.2:1.8:1.0:3.6)	Li et al. (2021)
12	ASPA	Man:Rha:GlcA:GalA:Glc:Gal:Xyl:Ara:Fuc (0.3:13.5:1.9:27:3.6:22.3:15.1:15.7:0.6)	Li et al. (2021)
13	ASPA-1	Man:Rha:GlcA:GalA:Glc:Gal:Xyl:Ara (0.2:13:0.8:35.9:0.6:17:25.3:7.2)	Li et al. (2021)
14	ASPA-1-A	Man:Rha:GlcA:GalA:Glc:Gal:Xyl:Ara:Fuc (0.4:14.8:1.1:26.9:0.6:20.4:27.1:8.3:0.4)	Li et al. (2021)
15	ASPA-1-B	Man:Rha:GlcA:GalA:Glc:Gal:Xyl:Ara:Fuc (0.4:11.7:0.9:46.2:1:14.1:20.4:5.1:0.2)	Li et al. (2021)
16	ASPA-2	Man:Rha:GlcA:GalA:Glc:Gal:Ara (0.4:11.4:1.8:15.7:2.2:44.7:23.8)	Li et al. (2021)
17	ASPN	Man:Rha:GlcA:GalA:Glc:Gal:Xyl:Ara:Fuc (4.3:4.5:1:1.7:25.7:32.9:7.1:22.6:0.2)	Li et al. (2021)
18	ESPS	Man:Rha:GlcA:GalA:Glc:Gal:Xyl:Ara:Fuc (1.5:13.6:0.6:14.9:17.1:24.2:11.6:15.4:1.1)	Li et al. (2021)
19	ASA-1P3A	Glc: Xyl: Gal (22.4:5.2:1)	Li et al. (2021)
20	ASA-1P3B	Glc: Xyl: Gal (7.6:19.4:1)	Li et al. (2021)

Ara, arabinose; Gal, galactose; Glc, glucose; GalA, galacturonic acid; GlcA, glucuronic acid; Man, mannose; Rha, rhamnose; Xyl, xylose; Fuc, fucose.

A recently published review compiled techniques of extraction and purification of polysaccharides from *E. senticosus* and structural characterization of these compounds, as well as the studies on their pharmacological effects (Li et al., 2021).

### 3.6 Terpenoids and other constituents of root essential oil

There are four reports on *E. senticosus* root essential oil composition (Yu et al., 2006; Richter et al., 2007; Załuski and Smolarz, 2015; Lim et al., 2007) (Table 3). The yield of volatile oil ranged from 0.05% (Yu et al., 2006) to 0.2% (Załuski and Smolarz, 2015). Gas chromatography with mass spectrometry was used to identify volatile compounds (Richter et al., 2007; Załuski and Smolarz, 2015; Lim et al., 2007) as well as its variants (Yu et al., 2006). In a study conducted by Yu *et al.*, sixty-eight compounds were identified (Yu et al., 2006). The content of 20 components was more than 1%. Caryophyllene oxide (16.7%) and isocaryophyllene (9.97%) were present in the highest amounts. Other compounds that occupied more than 5% of total volatile oils included β-farnesene, (*E,E*)-2,4-decadienal, and α-pinene. Another study reported that of 27 compounds identified from volatile oil from the root, the major ones were α-pinene (21.90%), and (+)-aromadendrene (3.77%) (Lim et al., 2007). Another analysis described by Richter *et al.* showed that samples of different origins had a different composition. For instance (*E*)-anethole was a major compound in two of three samples (27.9%, sample from Russia, and 17.2%, sample from China) and a minor one (1%) in the root collected in China. Thymol (4.7%) was present only in one sample originating from China (Richter et al., 2007). In a study by Załuski *et al.*, the major constituents included (*E,E*)-farnesol (33.7%) (*E,Z*)-farnesol (7.2%), and allo-aromadendrene (6.3%) (Załuski and Smolarz, 2015). Such diversity in the chemical profile of essential oils may be ascribed to ecological, climatic, or genetic factors, as samples came from different countries such as China (Yu et al., 2006; Richter et al., 2007), Korea (Lim et al., 2007), Russia (Richter et al., 2007), and Poland (Załuski and Smolarz, 2015). Richter *et al.* suggested the existence of different ‘chemotypes’, but this has not been previously described in this plant and requires further studies (Richter et al., 2007).

**TABLE 3:** Major volatile components (>10%) reported in the literature.

No.	Compound name	References
1.	(*E*)-anethole	Richter et al. (2007)
2.	α-pinene	Lim et al. (2007)
3.	Caryophyllene oxide	Yu et al. (2006)
4.	(*E,E*)-farnesol	Załuski and Smolarz (2015)

## 4 Quality of herbal preparations

Quality of plant material and commercial products was assessed in a few studies (Slacanin et al., 1991; Yat et al., 1998; Harkey et al., 2001; Han et al., 2016; Ruhsam and Hollingsworth, 2017; Maruyama et al., 2008; Zhu et al., 2011). Generally, the plant material cut and powdered may be identified by anatomic features using a simple microscopy method described in the Pharmacopoeia’s monographs. The quality control according to the description in the European Pharmacopoeia, the sum of eleutheroside B and eleutheroside E in *E. senticosus* dried whole or cut underground organs should not be less than 0.08% (European Pharmacopoeia, 2019). However, both geography and climate factors, such as temperature, precipitation, and relative humidity, affect the distribution of these compounds in *E. senticosus* root (Guo et al., 2019). Yan *et al.* examined 16 commercial samples of *E. senticosus* in the form of capsules, powder, and liquid. The content of eleutherosides B and E, analyzed by reversed phase HPLC, ranged from 0.01% to 1.0% and from 0.04% to 0.66% in the case of capsules and powder, respectively. The concentrations of eleutherosides B and E in liquid preparations were in the range of 0.03–0.13 mg/mL and 0.01–1.62 mg/mL, respectively (Yat et al., 1998).

Another study reported that the total concentration of eleutherosides B and E in 11 dietary supplements available in the United States varied 43-fold (0.041%–1.766% by weight) in the powdered samples and more than 200-fold (0.027–5.509 mg/mL) in the liquid extracts. In all analyzed products, the actual amount of eleutherosides did not match the labeled one, as it ranged from 11.9% to 327.7%. All dietary supplements tested contained *E. senticosus* (Harkey et al., 2001).

Another study assessed the authenticity of more than 1,400 samples representing nearly 300 different medicinal species from primary TCM markets in China. DNA barcoding was used to identify adulterant species. This technique is based on the concept that each member of any species has a unique short genomic sequence that does not occur in members of other sister species (Hebert et al., 2003; Bhargava and Sharma, 2013). The presence or absence of these diagnostic characters (referred to as taxon ‘barcodes’) allows species identification. Six *E. senticosus* samples were analyzed, except one sample that could not be successfully amplified. Two samples were found to be adulterant and identified as *Alangium chinense* (Lour.) Harms and *Aralia* sp. (Han et al., 2016).

Ruhsam *et al.* also used DNA barcoding methods to test 25 eleuthero supplements available in the United Kingdom market. Although all the tested preparations contained *E. senticosus*, about 36% also contained other members of the genus. These products were probably interchanged with *Eleutherococcus sessiliflorus* (Rupr. and Maxim.) which is distributed in the same regions and has been locally used as eleuthero root. The underground parts are extremely difficult to distinguish morphologically between these two species. Such substitution is improper since *E. senticosus* and *E. sessiliflorus* have different bioactive compounds (Ruhsam and Hollingsworth, 2017).

DNA sequence analyses were also conducted by Maruyama *et al.* to identify species of the plant material of 22 commercial samples available in the Japanese and Chinese markets. *Eleutherococcus senticosus* was present only in 68% of the samples. The plant material of 29% and 3% of the samples was probably *E. sessiliflorus* and *Aralia elata* var. *mandshurica* (Rupr. and Maxim.) J.Wen, respectively. Ultraperformance liquid chromatography (UPLC) combined with mass spectrometry was used for quantitative analysis of three selected markers: eleutheroside E, eleutheroside B, and isofraxidin. Eleutheroside E was present in samples made from *E. senticosus* as well as related species from the Araliaceae family, especially *E. sessiliflorus*. The authors indicate that eleutheroside B and isofraxidin can be used as marker compounds since they were detected in all samples containing *E. senticosus* (with a few exceptions) (Maruyama et al., 2008).

Two methods, such as analysis of chloroplast trnK intron sequences and PCR-restriction fragment length polymorphism (RFLP) assay, were used by Zhu *et al.* to discriminate other *Eleutherococcus* sp. in quality control of *E. senticosus* preparation. Of 40 commercial *E. senticosus* drugs available in Japanese markets, the plant material of 12 samples (30%) did not contain eleuthero, while two samples were of mixed sources. Replacing *E. senticosus* with *E. sessiliflorus* was found to be quite frequent and unsuitable in the context of the phytochemical profile and potential activity. Moreover, the quality of active compounds in tested preparations showed more than 10-fold difference in the total contents, while eleutheroside B and E ranged from 0.004% to 0.258% and 0.004%–0.127%, respectively. (Zhu et al., 2011).

Another not analysis of ten different commercial samples of *E. senticosus* root and three commercial *E. senticosus* tinctures showed some differences in qualitative and quantitative composition. Five samples originating from China had a higher syringin (eleutheroside B) content than eleutheroside E, while in those from Siberia, eleutheroside B was present in lower quantity. In the Korean sample concentration of eleutheroside B was below the limit of detection. Of the three tested tinctures, only one contained both eleutherosides (Slacanin et al., 1991).

**FIGURE 3:**
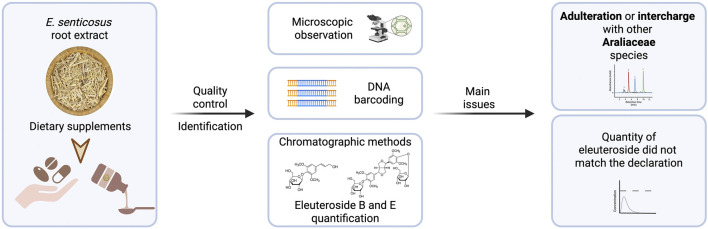
Assessment and main issues with quality control of *E. senticosus* and its preparations.

The studies mentioned above showed that there are many quality differences in commercial *E. senticosus* products. This poses a risk to consumers as such a supplement might not exert any effect or even cause adverse reactions. For example, there was a case of neonatal androgenization associated with maternal eleuthero use in Canada (Koren et al., 1990). Subsequent analysis demonstrated that the implicated material was from *Periploca sepium* Bunge, a toxic plant containing cardiac glycosides and pregnane-type steroids (Awang, 1996). In conclusion, proper identification and standardization of the plant material may be necessary to ensure the quality of herbal products (Figure 3).

## 5 Pharmacological activities

Predominantly, preparations of *E. senticosus* root are recommended for reducing fatigue, tiredness, loss of concentration, and stress, and for stimulation/modulation of the immune system to increase body resistance to cold, physical exhaustion, viruses, or bacteria. Most of the reported pharmacological activities are more or less connected with the above indication and often described as adaptogenic activities.

Adaptogens are natural substances that enhance the body’s nonspecific resistance to stress by modulating the ability to adapt and survive. One theory states that adaptogens’ action is related to their effects on the regulation of stress hormones and key mediators of homeostasis regulation (Panossian, 2017). The pleiotropic action of *E*. *senticosus* roots is believed to be due to its rich content of active compounds, mainly eleutherosides (lignan derivatives, coumarins, and phenylpropanoids-syringin) studied extensively for, among others, antioxidant, immunomodulatory, anti-inflammatory, anti-rheumatic, hepato-protective, hypolipidemic, or anticoagulant activity (Panossian and Wagner, 2005; Panossian and Wikman, 2009). In this review, we focus on the most popular, but at the same time not fully understood pharmacological properties of *Eleuthero coccus* roots which are the basis of their adaptogenic action– anti-inflammatory, immunomodulatory and neuroprotective activity (Figure 2). Resident microglia and macrophages are the main guardians of homeostasis, maintaining the immune balance of the headquarters nervous system (CNS) (Wang et al., 2020). Neuroinflammatory responses of microglial cells are associated with excessive production of inflammatory mediators, i.e., tumor necrosis factor TNF-α, interleukins (ILs), nitric oxide (NO), prostaglandin E2 (PGE2), and reactive oxygen species (ROS). Jung *et al.* presented that *Eleuterococcus* root extract application in concentrations 25 and 50 μg/mL was effective in the inhibition of iNOS (inducible nitric oxide synthase) in the murine macrophage RAW264.7 model, without an inhibitory effect on COX-2 expression (Kim et al., 2015; Jung et al., 2007). The same study confirmed the effects of *E*. *senticosus* extract on the inhibition of activation of Akt and MAPKs (JNK, ERK, and p38) in LPS-stimulated macrophages, indicating potential pathways of anti-inflammatory activity of this raw material. Interestingly, in Lin *et al.* study, *E. senticosus* stem bark extract used in concentrations 1–1,000 μg/mL (standardized to a minimum of 0.8% syringin and syringaresinol per 100 g of total extract) significantly inhibited iNOS expression already at a concentration of 10 μg/mL. Obtained results for stem bark extracts *in vitro* were also confirmed *in vivo* on the mouse peritoneal macrophages model (Lin et al., 2007; Lin et al., 2008). Although stem bark is mostly used in Asian medicines, the presence of similar compounds, especially syringin, at the concentration demanded by the European Pharmacopoeia for *Eleuterococcus* root, suggests the potential activity of root extract as well.

**FIGURE 4:**
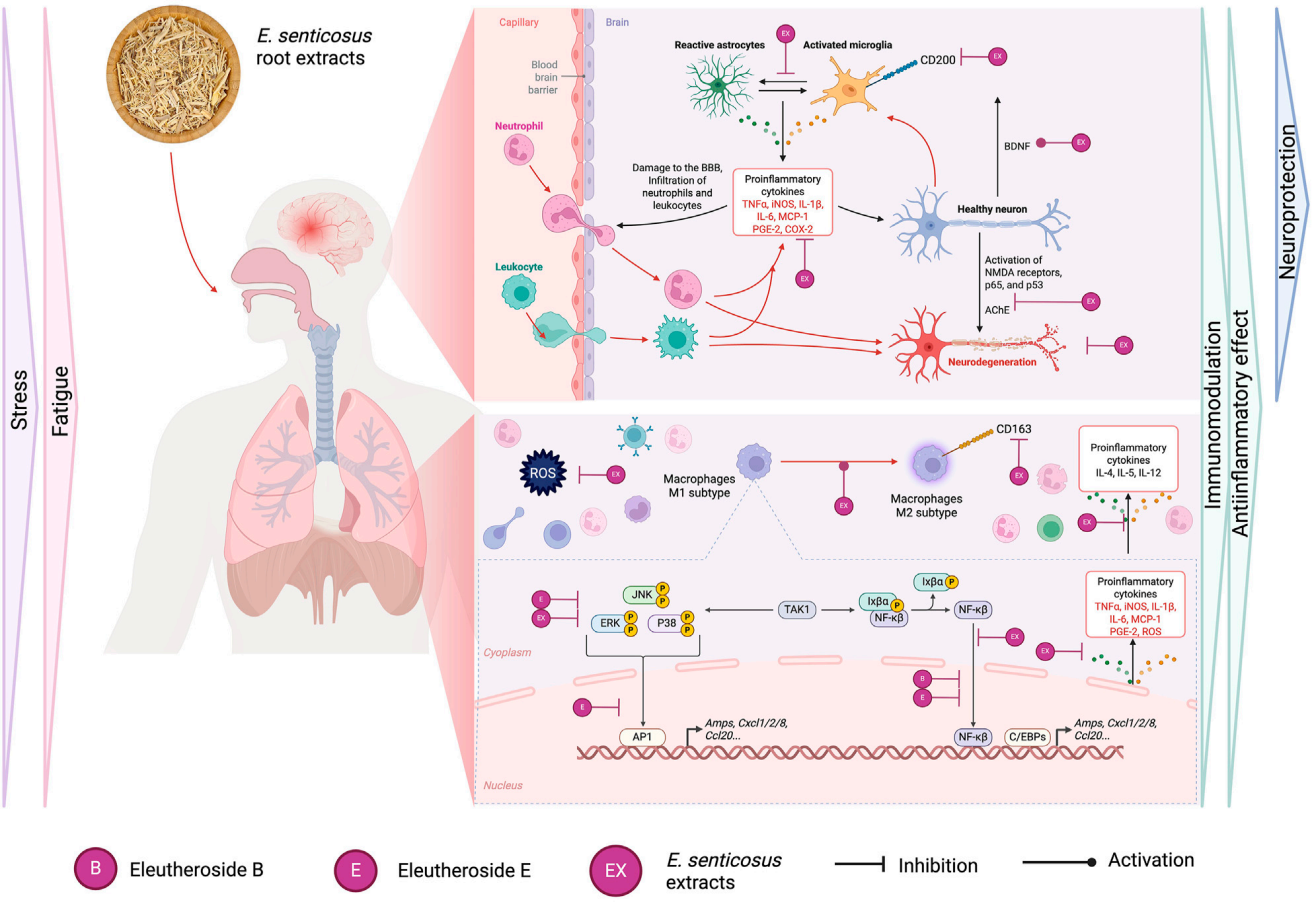
Summary of the pharmacological properties of *E. senticosus* roots and their leading active compounds in the context of potential activity after oral administration.

The pharmacological properties of *E. senticosus* roots and their leading active compounds described in this chapter are summarized in Figure 4.

An important anti-inflammatory role in the components of the *Eleutherococcus* root is also attributed to the presence of the polysaccharide fraction (ESPS). Many studies indicate a significant regulation of the secretion of inflammatory mediators (IL-2, IL-4, INF-γ) and the prevention of the formation of an inflammatory infiltrate in many *in vivo* models after the use of ESPS. Li *et al.* indicate inhibition of NF-κB activation in mouse liver tissue and prevention of LPS/D-GalN-induced endotoxic shock after ESPS administration (Li et al., 2021).

On the other hand, in line with previous reports, the main structures isolated from *E*. *senticosus* roots significantly inhibited iNOS and COX-2 expression in various cell models, e.g., eleutheroside B (12.5 and 25 μg/mL) in the IEC6 epithelial cell line, or isofraxidine (10–40 µM) in the nucleus pulposus (NPC) cell model (Wang et al., 2020; Zhang et al., 2020; Su et al., 2019). Moreover, these single compounds suppressed the gene expression of IL-6, MCP-1, INF-γ, and TNF-α, while only eleutheroside E inhibited transcription factors of NF-κB and AP-1 binding activities, which may indicate that it is the main compound responsible for the anti-inflammatory properties of this raw material (Figure 4) (Yamazaki et al., 2006; Yamazaki et al., 2004; Che et al., 2019; Wu et al., 2017).

However, the latest study by Tan *et al.* reports a significant effect of syringin (eleutheroside B) (10, 20 mg/kg) on the inhibition of NF-κB translocation to the nucleus, with a simultaneous inhibitory effect on the expression of NF-κB, IL-1β, IL-6, TNF-α, myeloperoxidase (MPO) and the promotion of FOXO3a phosphorylation in a rat model of cerebral ischemic injury (Tan et al., 2021). The anti-inflammatory effects of eleutheroside B were also confirmed earlier in the mouse model of RAW264.7 LPS-stimulated cells. However, significant inhibition of TNF-α in this model was effective only at a concentration of 125 μM; for comparison, the dexamethasone control inhibited TNF-α production already at a concentration of 0.1 µM (Cho et al., 2001). The lack of activity of the root extract against COX-2 could result from the used concentrations (25, 50 μg/mL) and/or the lack of standardization for the content of individual compounds in the tested extract (Yamazaki TM et al., 2006; Jung et al., 2003). However, it should be noted that the mouse immune cell model does not fully reflect the immune response in the human organism.

The first preliminary report on the effect of ethanolic extracts of *E. senticosus* roots in a model of human whole blood obtained from healthy volunteers was conducted by Schmolz et al. Blood treatment with *E. senticosus* root extracts over a wide range of dilutions in cell culture (1:125; 1:500; 1:2,000; 1:8,000; 1:32,000), showed the stimulation of Rantes secretion while inhibiting the production of IL-4, IL-5, and IL -12. Moreover, in the full range of concentrations, *E. senticosus* extracts could increase or decrease the concentration of G-CSF, IL-6, and IL-13, which indicates their potentially dose-dependent immunomodulatory effect (Schmolz et al., 2001). On the other hand, Jin *et al.*, for the first time, analyzed the effect of *E. senticosus* root extracts (not standardized for the content of active ingredients) on the activity of human macrophages isolated from peripheral blood. The novelty of this study was the simultaneous comparison of the bark and root extracts (10–30 μg/mL) in a model of unstimulated macrophage cells, pointing to the potential immunomodulatory effect of the whole extracts. Both root and bark extracts were shown to inhibit CD163 receptor expression in a concentration-dependent manner on the surface of macrophages, which was associated with a likely effect on the mTORC2 pathway (Jin et al., 2020). In the same study, *Eleutherococcus* root and bark extracts were shown to be active in promoting actin polymerization, increasing migratory capacity and phagocytosis of *E. coli*, which the authors of the paper attributed to an indirect effect on the polarization of macrophages towards the anti-inflammatory M2 subtype. Furthermore, the root extract, in contrast to the bark extract, increased IL-4-induced expression of the CD200R related to controlling inflammatory reactions and neurodegenerative symptoms. Interestingly, CD200 signaling was close to zero in models of ischemic stroke (Ritzel et al., 2019), while in an *in vivo* study, reduced expression of neuronal CD200 was observed in the hippocampus of aged and β-amyloid treated animals, providing evidence that neurons may reduce microglial activation through CD200 signaling (Ngwa and Liu, 2019). In the study conducted by Lee et al., administration of *E. senticosus* stem bark extract (0.486% ± 0.046% of eleutheroside E) at doses of 30 and 300 mg/kg attenuated ischemia-induced upregulation of COX-2 expression, as well as the activation of astrocytes and microglia in the CA1 region of the hippocampus (Lee et al., 2012). However, there are no more recent reports confirming this observation, and the use of stem bark extract may only partly support the root activity.

In a non-LPS-stimulated cell model, the bark extract, but not the root extract, induced the p38 MAPK pathway, significantly affecting the production of TNF-α and anti-inflammatory IL-10. It is also worth noting that the differences in presented effects may have been due to differences in the chemical composition of the extracts from the root, rich in syringin and caffeic acid, and the bark, with an additional significant sesamin content (Jin et al., 2020; Bain and Mowat, 2012).

Support of the immune system by *Eleutherococcus* root may also be expressed through the protection of the body against immunomodulatory and toxic substances, i.e., heavy metals. In an *in vivo* study, long-term administration of *E. senticosus* extract in combination with high doses of CdCl_2_ (an intoxicating agent) led to a significant reduction of cadmium concentrations in the blood and liver compared to a group of control mice, furthermore reducing cadmium-induced mitotic and apoptotic activity of liver cells (Smalinskiene et al., 2009). The detoxifying properties of *E. senticosus* have been exploited in Korea, where *E. senticosus* extract is used as an ingredient in traditional Korean herbal medicine and is available as a functional beverage to reduce liver damage and accelerate alcohol detoxification (Park et al., 2006).

The important role of the NF-κB pathway is seen not only as a therapeutic target in the treatment of neurodegenerative diseases associated with neuritis but also in acute inflammation seen in myocardial infarction (MI), acute lung injury, or metabolic disorders (Frantz et al., 2007). Considering the indications for the use of *E. senticosus* root as a tonic agent (Huang et al., 2011a), Wang *et al.* investigated the effect of eleutheroside E, the lead compound in *E. senticosus* roots, on H9c2 cardiac cell line, assuming the protective effects of eleutheroside against MI/R injury. Eleutheroside E in the adopted model inhibited cell apoptosis depending on the concentration (30, 60, 100 µM) and reduced cell death, which was indicated by lower production of lactate dehydrogenase (LDH) and High mobility group box 1 protein (HMBG1). Relating to the NF-κB signaling pathway, eleutheroside E at a concentration of 100 μM, significantly inhibited NF-κB activation and ERK, JNK, and p38 phosphorylation in this model. Disruption of NF-κB translocation decreases the release of pro-inflammatory cytokines, which in this case was expressed in reduced production of IL-6, TNF-α after eleutheroside E treatment. The above results indicate that this compound-mediated activation of NF-κB probably requires the involvement of three MAPKs at the same time in the infarction model (Wang and Yang, 2020). These observations were partially supported by Fei *et al. in vivo*, where *E. senticosus* root extract at the dose of 20 mg/kg, reduced IL-6 and TNF-α secretion in a mouse model of LPS-induced acute lung injury by inhibiting the NF-κB signaling pathway (Fei et al., 2014). Conversely, the research conducted by Zhang et al., which integrates both *in vitro* experiments and molecular docking analyses, suggests that *E*. *senticosus* extracts may exert a multi-targeted effect in mitigating anxiety and memory deficits associated with Alzheimer’s disease. These effects are primarily mediated through the phosphorylation and activation of the MAPK signaling pathway, with the main targets including APP, NTRK1, EGFR, GSK3B, and other related genes (Zhang et al., 2024).

The primary mechanism of *E. senticosus*, responsible for the potential neuroprotective effect, has not yet been fully elucidated. Bai Y. *et al.* indicate that fractions of ethyl acetate, n-butanol, and water, derived from methanolic extracts of *E. senticosus* roots showed significant protective effects at 1, 10, and 100 μg/mL, against Aβ-induced neurite atrophy in a model of rat cultured cortical neurons. The particular activity was noted in fractions rich in eleutheroside B, E, isofraxidine, and β-sitosterol glycosides. The activity of the tested compounds showed statistical significance at low concentrations (1 and 10 µM), which may indicate their actual therapeutic efficacy on neuronal morphological plasticity under neurodegenerative conditions (Bai et al., 2011). These protective properties towards axons and neurons had already been demonstrated a few years earlier for eleutheroside B in a study conducted by Tohda *et al.* (also in the concentrations 1–10 µM) (Tohda et al., 2008). Particularly noteworthy is the observation of Song *et al.*, who showed that the bioavailability of eleutheroside E and syringin in brain tissues is relatively higher than that of the other constituents, which may indeed explain its major importance in neuroprotection and regulation of cognitive functions (Song et al., 2022).

A study by Adamczyk et al. demonstrated the inhibitory potential of a 75% methanolic extract of *E. senticosus* root on anti-acetylcholinesterase (AChE) degradation to 26.1% ± 0.05% (Adamczyk et al., 2019). In another study, where 12 extracts from six *Eleutherococcus* species were tested, extracts from *E. setchuenensis* and *E. sessiliflorus* showed the strongest, though moderate, inhibition of AChE activity (IC_50_: 300 and 300 μg/mL, respectively). In comparison, *E. senticosus* root extract had an IC value of 460 ± 40 μg/mL (Załuski and Kuźniewski, 2016). Huang *et al.* indicated a dose-dependent increase in acetylcholine and a decrease in choline content after treatment with eleutherosides, suggesting an increased reutilization of choline, thereby accelerating acetylcholine synthesis. The present study used a rat model of ageing induced by the injection of quinolinic acid into the hippocampal area CA1. The used doses of eleutherosides B and E (50, 100, 200 mg/kg) in a randomized animal model yielded a broad cross-section of the activity of the tested compounds, presenting dose-dependent changes in the Morris maze test and the acetylcholine and choline content of the tested hippocampal homogenates. This effect may correlate with a potential increase in synapse plasticity and mitigation of hippocampal neuronal damage (Huang et al., 2013).

Moreover, the hippocampus is involved in learning, memory, as well as chronic stress and depressive-like behaviours (Price and Duman, 2020). Anxiety and depressive disorders observed in rats after myocardial infarction were strongly correlated with decreased brain-derived neurotrophic factor (BDNF) levels in the hippocampus. BDNF plays an important role in neurogenesis and synapse plasticity, as well as cognitive function and mood. In a study by Kiyazaki *et al.*, administration of a 5% aqueous extract of *E. senticosus* root standardized for the content of isofraxidine, eleutheroside B, eleutheroside E, eleutheroside B1, and chlorogenic acid, variably increased the amount of BDNF mRNA in the hippocampus of rat models (Miyazaki et al., 2019). The effect of *E. senticosus* on the regulation of BDNF expression in the hippocampus may justify the antidepressant mechanism of *E. senticosus*, as well as the effects of memory and concentration improvement. The most recent study, on Alzheimer’s disease (AD) rat model, revealed that *E. senticosus* root extract at doses of 300 and 600 mg/kg improve cognitive function and alleviate AD-like pathological features in the Morris water maze test (Yang et al., 2025). Thhe extract activity was connected with the activation of the Nrf2 (Nuclear factor erythroid two-related factor) signaling pathway, thereby protecting the brains from oxidative stress (Yang et al., 2025). Several studies have shown that oxidative stress, in addition to increasing inflammation, may also be closely linked to the pathogenesis of neurodegenerative diseases (He et al., 2020; Liu et al., 2018; Zgórka and Kawka, 2001). Under pathogenic conditions of infection, stress, or drug exposure, the production of reactive oxygen species (ROS): superoxide anions, hydrogen peroxide, and hydroxyl radicals or free radicals can be enhanced, leading to the oxidation of lipids, proteins, and DNA (Park et al., 2006; Su et al., 2022). In studies of antioxidant components in different parts of *E. senticosus*, the presence of a significant proportion of metabolites positively correlated with oxidative stress was confirmed in the roots compared to the seeds and leaves. Furthermore, it appears that the phenolic acids (gentisic acid, caffeic acid), vanillin, coniferolaldehyde, and lignans (matairesinol (+)-lirioresinol B, acanthoside B, eleuteroside E) contained in the roots of *E*. *senticous* have a higher anti-radical capacity than the active substances predominant in the leaves and seeds (Su et al., 2021). In a study by Kim Y. *et al.*, a significant antioxidant effect was also attributed to chlorogenic acid activity (Kim et al., 2015).

The antioxidant activity of *E. senticosus* roots was confirmed, among others, in an H_2_O_2_-treated PC12 cell model, as well as in H_2_O_2_-RAW264.7 and DSS mouse cell models (Su et al., 2022; Su et al., 2021). In an *in vitro* model, *E. senticosus* flavonoids (structures not specified) effectively improved (in a dose-dependent manner) the ability of macrophages to eliminate ROS and also increased the capacity of antioxidant enzymes (CAT, SOD, GPx) in H_2_O_2_-stimulated RAW264.7 cells, increasing antioxidant capacity (Su et al., 2022). Inhibition of ROS production was also demonstrated for eleutheroside E in the previously mentioned study by Wang *et al.* in the H9c2 cell model at a quite high concentration of 100 µM (Wang and Yang, 2020).

Underlying the adaptogenic action of *Eleutherococcus* root is a combination of anti-inflammatory, immunomodulatory, and antioxidant activity, which may contribute to the concentration- and memory-enhancing effects following the use of root preparations. Eleutherosides B, E, and isofraxidine appear to be of particular importance in modulating the adaptogenic response. However, there are opinions that the best effect is due to the synergistic action of all these constituents from the whole, unfractionated raw material (Załuski and Kuźniewski, 2016; Załuski et al., 2018).

## 6 Human studies and clinical trials

Surprisingly, there are not many human or clinical studies of sufficient quality to confirm the beneficial effects of *Eleutherococcus* root preparation on the human body. This may be due, among other, to the fact that it was not very popular outside the former Soviet Union. The Soviet Union was a pioneer in the use of eleuthero, so it was natural to look for research results on its efficacy in that country. Gerontakos et al. (2021) published in 2021 a very comprehensive review of clinical trials conducted during the time of the Soviet Union. The undoubted advantage of these studies is the often very large number of participants (usually large companies and industrial plants). On the other hand, the results of these studies are subject to considerable limitations, especially in terms of methodology, e.g., randomisation, control group, statistical analysis methods, or lack of placebo. Many details of the study participants were often missing from the reports of these studies. Nevertheless, among the results analysed by the authors, there are some that may confirm the efficacy of eleuthero in certain conditions, or at least provide a basis for designing and conducting contemporary clinical trials according to modern standards.

Among the Soviet studies described by Gerontakos et al. (2021), particularly noteworthy are those on the effects on cognitive function, physical performance, vision, hearing, but also the prevention of seasonal diseases, cardiovascular conditions, and the effects on pregnancy (!). Studies on cognitive function have yielded heterogeneous results on cognitive parameters, which appear to be dose-dependent. In all studies on the effects on physical performance under normal conditions and under extreme conditions, Eleuthero preparations administration was found to lead to improved work capacity, increased physical endurance, reduced recovery times, and increased resistance to fatigue. *Eleutherococcus senticosus* was also found to have a beneficial effect in the prevention of influenza and seasonal respiratory infections, both alone and in combination with conventional treatment. Contradictory results were observed for the effect of *E. senticosus* on cholesterol levels, with various studies reporting both a decrease, no effect, and even an increase in cholesterol levels. On the other hand, there was a general improvement in symptoms in patients with atherosclerosis, such as chest pain, shortness of breath, and fatigue. The same was true for the effect on blood pressure: both a significant reduction was observed in people with hypertension, normalization of blood pressure in healthy study participants during physical work at high altitudes, but in the same study, an increase in blood pressure was also observed in some people with hypertension. However, the lack of detailed data on the number of participants who experienced an increase in blood pressure does not allow to determine whether hypertension may be a contraindication to the use of eleuthero preparations.

In addition, one clinical study observed a beneficial effect of *E. senticosus* on visual function, and two others showed some benefits in hearing disorders. In turn, in several studies, the administration of eleuthero preparation to oncology patients during chemotherapy resulted in an improvement in general wellbeing, including appetite, sleep, mood, and some biological parameters.

However, more recent clinical studies do not unequivocally confirm the beneficial effects of *Eleutherococcus* root preparations.

### 6.1 Asthenia, stress and chronic fatigue

The beneficial effects of *E. senticosus* in stress adaptation were demonstrated in a study on a group of 45 young, healthy volunteers (Facchinetti et al., 2002). Study participants received eleuthero preparation (two vials per day - no information about method of extraction nor concentration) or placebo for 30 days. The Stroop Colour-Word test was used as a challenge stressor before and after treatment. Before treatment subjects reacted to the stimulation with an increase in both systolic blood pressure and heart ratio. It has been observed that treatment with *E*. *senticosus* resulted in a reduces cardiovascular stress response in comparison to placebo. Interestingly, female participants seem to benefit more than males from its adaptive properties.

However, in a randomized placebo-controlled trial conducted on a group of 96 people, including volunteers and members of chronic fatigue support group, *E. senticosus* extract standardized for eleutherosides B and E content (4 capsules containing 500 mg of extract per day) or placebo was administered for 2 months (Hartz et al., 2004). Subsequently, for a further 2 months, participants received only the extract as a reward for participating in the study. After 2 months of administration of the extract, a greater improvement in the Rand Vitality Index was observed in the group receiving the eleuthero extract compared to the placebo group, but this was not statistically significant. Only for subjects with moderately fatigue at baseline there was a statistically significant improvement observed compared to placebo.

Another randomized controlled study was conducted to compare the effect of 120 mg/day of *E*. *senticosus* root extract, 2 days of professional stress management training and a combination of both (Schaffler et al., 2013). The 8-week study involved 144 participants suffering from asthenia and reduced working capacity related to chronic stress. Authors measured parameters such as cognitive performance; feelings of stress, fatigue and exhaustion; alertness, rest, and mood; quality of life and sleep; physical complaints and physical activity; and physiological stress parameters, including cortisol awakening response. While statistically significant differences in some measured parameters were observed, such as mental fatigue improved more in group with combination therapy compared to other groups as well as calmness-restlessness revealed superiority of combination therapy over *E*. *senticosus* group with the duration of the study, the conclusions of the study showed no beneficial effects of adding regular intake of *E*. *senticosus* to stress management training on subjective wellbeing, cognitive tests, as well as physiological markers of stress in individuals experiencing high levels of stress and associated reduced work performance, ability to concentrate, fatigue.

Interesting results were obtained in a pilot clinical trial (Phase IIa) of a complex preparation containing standardized extracts of *Rhodiola rosea* L., *Schisandra chinensis* (Turch.) Baill. and *Eleutherococcus senticosus* Maxim (ADAPT-232) (Aslanyan et al., 2010). The amounts of the main active ingredients in the preparation were determined: rhodioloside, tyrosol, rosavin, eleutheroside B, eleutheroside E, schizandrin, and γ-schizandrin. It was a double-blind, placebo-controlled, randomized trial with two parallel groups. Forty healthy women between the ages of 20 and 68 were selected to participate in the pilot study, claiming to have experienced long-term stress due to living under psychologically stressful conditions. Participants received either a single tablet of ADAPT-232 (270 mg), or a single placebo tablet. The effect of the preparation was measured before treatment and 2 hours after treatment using the d2 Test of Attention used to assess patients’ cognitive function. The d2 test measures selective attention and is sensitive to the speed and quality of performance. The results obtained in the study showed that ADAPT-232 significantly improved scores resulting from the psychometric test, in comparison to placebo. Subjects taking ADAPT-232 experienced an increase of the amount of precise/correct work completed on the background of mental fatigue, and their ability to focus, processing speed, and accuracy of performing specific tasks improved. These effects were seen 2 hours after intake of ADAPT-232. No serious adverse effects were reported. However, due to the complexity of the formulation, the beneficial effect cannot be clearly attributed to any of the ingredients (McNaughton et al., 1989).

### 6.2 Endurance exercise

Since adaptogens are also popular as natural endurance enhancers in athletes, the effect of *Eleutherococcus* root on endurance performance has been studied.

In a study by Szolomicki (Szołomicki et al., 2000) on cellular defense and physical fitness in man, conducted on 50 healthy volunteers, the effect of administering an ethanolic extract of eleutheroroots (1 g fluid extract equivalent to 1 g root) for 1 month (25 drops three times daily) was compared with a preparation of the herb *Echinacea purpurea* (L.) Moench. Those taking *E. senticosus* root extract showed a decrease in total cholesterol, LDL, and free fatty acids (FFA) levels after 1 month of treatment, as well as an increase in phagocytic activity of neutrophils measured as Wright’s index. Beneficial effects were also observed on parameters used to evaluate physical fitness: a significant rise in maximal oxygen consumption per body mass and heart rate. Other parameters remained unchanged. These results, although promising, are of limited value due to the lack of a placebo and randomization of groups.

In contrast, studies by Dowling et al. (1996) and McNaughton et al. (1989) found no beneficial effects of eleuthero root preparations on performance. Both double-blind, placebo-controlled studies of 6 weeks each involved trained runners. In the first study, in which 30 participants received 1,000 mg of extract (no information about extraction and standardization) daily, an increase in pectoral and quadriceps strength was observed; however, the use of this preparation did not increase maximal oxygen consumption or affect HR recovery. In the second, 20 participants received 3.4 mL of an ethanolic extract (no information about extraction and standardization) of eleuthero root daily for 6 weeks. A running test was performed every 2 weeks, as well as 2 weeks after the end of supplementation, and the parameters oxygen uptake, expired minute ventilation, ventilatory equivalent for oxygen uptake, and ventilatory exchange ratio were measured; heart rate was also monitored. The observed differences between the *E. senticosus* and placebo groups were not statistically significant, but the authors note that statistical power ranged from 0.16 to 0.38, which may indicate a type II statistical error. Both of these studies were conducted on a small number of participants.

Interesting results questioning the efficacy of *E. senticosus* compared to *Panax ginseng* were obtained in a clinical trial on competitive club-level endurance athletes engaged in their normal in-season training. Participants received ethanolic extract containing either *E. senticosu*s, *Panax ginseng* (equivalent to 4 g and 2 g/day of dried root, respectively), or placebo for 6 weeks. Effects on immune system parameters and steroidal hormone indices of stress were studied. While no significant changes in lymphocyte number were observed in either group, significant differences were observed in the group receiving *E. senticosus*: a decrease in the testosterone to cortisol ratio was observed, which was not observed in the other groups. This result suggests that *E. senticosus* not only does not decrease but rather increases hormone indices of stress (Gaffney et al., 2001).

### 6.3 Upper respiratory tract infection

A number of papers have been published on clinical trials of preparations containing eleuthero root extracts in upper respiratory tract disorders. Although the beneficial effects of these preparations have been demonstrated, it is difficult to attribute a special role to the presence of *E. senticosus* in them, given its relatively low percentage content in the preparations studied. Thom and Wollan (1997) studied the therapeutic effect of a mixture containing (per 100 mL) *Echinacea pallida* root (10 g), *Adathoda vasica* leaf (5 g), *E. senticosus* root (5 g), and *Glycyrrhiza glabra* root (5 g) in the treatment of uncomplicated upper respiratory tract infections (the common cold) in a randomized, double-blind, placebo-controlled parallel group clinical trial. The results of the study indicate that the mixture has advantages over a placebo in the treatment of uncomplicated upper respiratory tract infections. The number of days needed for treatment, as assessed by the patients themselves, was significantly reduced in the active group (5.2 days) compared to the placebo group (9.2 days). Narimanian et al. (2005a) compared a fixed combination of standardized extracts of *E. purpurea*, *Adhatoda vasica,* and *E. senticosus*, with the combined extracts of *E. purpurea* and *E. senticosus* alone (Echinacea mixture) in a controlled, double blind, randomized trial, and with bromhexine (a standard treatment) in a controlled, open, randomized clinical trial on patients with non-complicated acute respiratory tract infections. The severity of coughing, frequency of coughing, efficacy of mucus discharge in the respiratory tract, and nasal congestion showed statistically greater improvement in patients treated with both herbal preparation compared with those receiving the standard treatment. However, the authors focused on the role of *Adhatoda vasica* in the efficacy of the formulation. The same researchers also demonstrated the beneficial effect of another *E. senticosus*-containing preparation in a double-blind, placebo-controlled, randomised pilot phase three study (Narimanian et al., 2005b). The study involved patients suffering from acute non-specific pneumonia treated with standard antibiotic therapy. The study group received as an adjuvant a preparation containing extracts of *R. rosea*, *S. chinensis,* and *E. senticosus*. The primary outcome measures were the duration of antibiotic treatment associated with clinical symptoms of the acute phase of the disease, together with mental performance scores on a psychometric test and self-assessed quality of life (QOL) before treatment and on the first and fifth day after clinical recovery. It was shown that the mean duration of antibiotic treatment required to recover from the acute phase of the disease was 2 days shorter in patients treated with the study preparation compared with patients in the placebo group. Barth et al. (2015) demonstrated beneficial antitussive effects of a combination of *J. adhatoda*, *E. purpurea,* and *E. senticosus* extracts in patients with acute upper respiratory tract infection in a comparative, randomized, double-blind, placebo-controlled study, but the paper focuses on the role of the antitussive effect of the vasicine alkaloid present in *Justicia adhatoda*. Melchior et al. (2000) presented the results of a double-blind, placebo-controlled study of the activity of standardised *Andrographis paniculata* extract in combination with *E. senticosus* in the treatment of uncomplicated upper respiratory tract infection. The study has shown that the tested combination reduces the typical symptoms of uncomplicated upper respiratory tract infection, but the authors focused mainly on the role of *A. paniculata* in the beneficial therapeutic effect, neglecting the role of *E. senticosus*, which accounted for about 10% of the active ingredients. Similarly, Gabrielian et al. (2002) studied the efficacy of the same preparation in a double-blind, placebo-controlled, parallel-group clinical trial for the treatment of acute upper respiratory tract infections, including sinusitis. The authors showed a favourable effect of the preparation compared with a placebo, also for sinusitis. Also, for this study, the authors attribute the beneficial effect of the *A. paniculata* preparation, ignoring the possible effect of *E. senticosus*. All of the studies mentioned, despite being concerned with preparations containing *E. senticosus* and standardized for, among other things, eleutherosides B and E, tend to focus on the role of the other ingredients in their efficacy and do not clarify the role of *E. senticosus* in the treatment of upper respiratory tract infections. It is also worth noting the relatively low *E. senticosus* content of these preparations.

**FIGURE 5:**
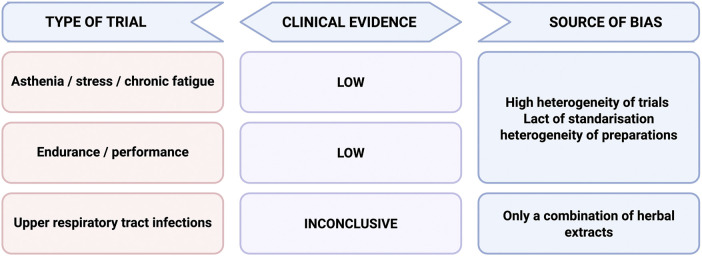
Efficacy of *E. senticosus* preparations-source of bias.

In summary, there are clinical studies both confirming the beneficial effect of eleuthero as an adaptogenic agent and those in which this effect has not been confirmed. Due to different criteria for the selection of study groups, different methods of assessing efficacy, it is not possible to assess its effectiveness unequivocally (Figure 5). It is difficult to answer whether *E. senticosus* is effective as an adaptogen, especially in comparison with other preparations of plant origin, such as *Panax ginseng* or *Rhodiola rosea*.

## 7 Conclusion and further scope

Various preparations of *E. senticosus* root, mostly food supplements, are available on the worldwide market as adaptogen, to reduces fatigue and stress, decreases blood glucose levels, and stimulates the immune system. The European Medicines Agency (EMA) approved *E. senticosus* root for the treatment of symptoms of asthenia, such as fatigue and weakness. The pharmacological effect is connected with various constituents such as phenolic acids and their derivatives, lignans, coumarins, triterpenoid saponins and polysaccharides. Caffeoylquinic acids prevail among the phenolic acids in *E. senticosus.* Syringaresinol and pinoresinol derivatives are the most frequently found lignans. Two marker compounds, namely eleutheroside B (syringin) and eleutheroside E (syringaresinol diglucoside), are used to standardize the eleuthero root products. Especially, syringin and in lesser extent isofraxidin 7-*O*-glucoside (eleutheroside B1), appear as a specific markers for discrimination from other Araliaceae plant materials. Interestingly, eleutheroside B is a quite rare natural compounds, with significant biological activity. The tools for quality control are well established and specific enough for *E. senticosus* discrimination, and should be applied for routine analysis. Unfortunately, this is not always a case for diet supplements and as the results the poor quality of preparation may strongly influence the efficacity of this interesting medicinal plant.

The preclinical studies, although mostly *in vitro* and using simplified models, demonstrated that the adaptogenic action of eleuthero root is a combination of anti-inflammatory, immunomodulatory, antioxidant and neuroprotective activity, which may contribute to the concentration- and memory-enhancing effects. The mechanism of action seems to be connected with inhibition of MAPKs, Akt and NF-κB activation, and increase of brain -derived neurotrophic factor (BDNF). Eleutherosides B, E, and in lesser extend isofraxidine appear to be of particular importance in modulating those adaptogenic responses. Not surprisingly, there are opinions that the best effect is due to the synergistic action of all these constituents from the whole, unfractionated raw material. However, more studies, especially those bioactivity-guided, are needed to identify and isolate more compounds from *E. senticosus*. Furthermore, the *in vivo* activity of isolated metabolites should be assessed to resolve the question of the active constituents of *E. senticosus*. More, *in vivo* studies connected with antifatigue, stress reduction and cognitive function are needed, especially as we observe no much more recent articles. We observed more publications concerning the bioactivity of other part of this plant (leaves, fruits) or other species of *Eleutherococcus* genus, and investigation of other biological activity (e.g. hepatoprotective or anti-diabetic effect).

The main issue is the lack of robust clinical evidence for the treatment of symptoms of asthenia as adaptogen. The high heterogeneity (e.g. different preparations, different methods of assessing efficacy) and low quality of clinical trial (e.g. non-characterized extracts, small groups) in connection with lack of proper standardization of *E. senticosus* preparations make impossible to assess the effectiveness, especially in comparison with other preparations of plant origin, containing *Panax ginseng* or *Rhodiola rosea.* Unfortunately, we can observe a vicious circle: preparations are of low quality what strongly influence the statistical significant of clinical observations, and this lead to the discontinuation of further research and make a production of high quality preparation unprofitable.

## References

[B1] Abbai R. Mathiyalagan R. Markus J. Kim Y. J. Wang C. Singh P. (2016). Green synthesis of multifunctional silver and gold nanoparticles from the oriental herbal adaptogen: Siberian ginseng. Int. J. Nanomedicine. 11, 3131–3143. 10.2147/IJN.S108549 27468232 PMC4946861

[B2] Adamczyk K. Olech M. Abramek J. Pietrzak W. Kuźniewski R. Bogucka-Kocka A. (2019). Eleutherococcus species cultivated in Europe: a new source of compounds with antiacetylcholinesterase, antihyaluronidase, anti-DPPH, and cytotoxic activities. Oxid. Med. Cell. Longev. 2019, 8673521. 10.1155/2019/8673521 30984341 PMC6431473

[B4] Aslanyan G. Amroyan E. Gabrielyan E. Nylander M. Wikman G. Panossian A. (2010). Double-blind, placebo-controlled, randomised study of single dose effects of ADAPT-232 on cognitive functions. Phytomedicine 17, 494–499. 10.1016/j.phymed.2010.02.005 20374974

[B5] Awang D. V. (1996). Siberian ginseng toxicity may be case of mistaken identity. CMAJ 155, 1237. 8964001 PMC1335056

[B6] Bai Y. Tohda C. Zhu S. Hattori M. Komatsu K. (2011). Active components from Siberian ginseng (*Eleutherococcus senticosus*) for protection of amyloid β(25-35)-induced neuritic atrophy in cultured rat cortical neurons. J. Nat. Med. 65, 417–423. 10.1007/s11418-011-0509-y 21301979

[B7] Bain C. C. Mowat A. M. (2012). CD200 receptor and macrophage function in the intestine. Immunobiol 217, 643–651. 10.1016/j.imbio.2011.11.004 22204814

[B8] Baranov A. I. (1982). Medicinal uses of ginseng and related plants in the Soviet Union: recent trends in the Soviet literature. J. Ethnopharmacol. 6, 339–353. 10.1016/0378-8741(82)90055-1 7154701

[B9] Barth A. Hovhannisyan A. Jamalyan K. Narimanyan M. (2015). Antitussive effect of a fixed combination of *Justicia adhatoda*, *Echinacea purpurea* and *Eleutherococcus senticosus* extracts in patients with acute upper respiratory tract infection: a comparative, randomized, double-blind, placebo-controlled study. Phytomedicine 22, 1195–1200. 10.1016/j.phymed.2015.10.001 26598919

[B10] Bhargava M. Sharma A. (2013). DNA barcoding in plants: evolution and applications of *in silico* approaches and resources. Mol. Phylogenet. Evol. 67, 631–641. 10.1016/j.ympev.2013.03.002 23500333

[B11] Bleakney L. T. (2008). Deconstructing an adaptogen: *Eleutherococcus senticosus* . Holist. Nurs. Pract. 22, 220–224. 10.1097/01.HNP.0000326005.65310.7c 18607235

[B12] Brekhman I. I. Dardymov I. V. (1969). New substances of plant origin which increase nonspecific resistance. Annu. Rev. Pharmacol. 9, 419–430. 10.1146/annurev.pa.09.040169.002223 4892434

[B13] British Pharmacopoeia Commission (2022). British pharmacopoeia. The Stationery Office.

[B14] Che D. Zhao B. Fan Y. Han R. Zhang C. Qin G. (2019). Eleutheroside B increase tight junction proteins and anti-inflammatory cytokines expression in intestinal porcine jejunum epithelial cells (IPEC-J2). J. Anim. Physiol. Anim. Nutr. Berl. 103, 1174–1184. 10.1111/jpn.13087 30990939

[B15] Cho J. Y. Nam K. H. Kim A. R. Park J. Yoo E. S. Baik K. U. (2001). *In-vitro* and *in-vivo* immunomodulatory effects of syringin. J. Pharm. Pharmacol. 53, 1287–1294. 10.1211/0022357011776577 11578112

[B16] Choi H. R. Nam K. M. Lee H. S. Yang S. H. Kim Y. S. Lee J. (2016). Phlorizin, an active ingredient of *Eleutherococcus senticosus*, increases proliferative potential of keratinocytes with inhibition of MiR135b and increased expression of type IV collagen. Oxid. Med. Cell Longev. 2016, 3859721. 10.1155/2016/3859721 27042261 PMC4799823

[B18] Davydov M. Krikorian A. D. (2000). *Eleutherococcus senticosus* (Rupr. and Maxim.) Maxim. (Araliaceae) as an adaptogen: a closer look. J. Ethnopharmacol. 72, 345–393. 10.1016/s0378-8741(00)00181-1 10996277

[B19] Deyama T. Nishibe S. Nakazawa Y. (2001). Constituents and pharmacological effects of *Eucommia* and Siberian ginseng. Acta Pharmacol. Sin. 22, 1057–1070. 11749801

[B20] Dowling E. A. Redondo D. R. Branch J. D. Jones S. McNabb G. Williams M. H. (1996). Effect of *Eleutherococcus senticosus* on submaximal and maximal exercise performance. Med. Sci. Sports Exerc. 28, 482–489. 10.1097/00005768-199604000-00013 8778554

[B22] ESCOP (2003). ESCOP monographs. In: European scientific cooperative on phytotherapy and thieme exeter. Argyle House, Exeter.

[B21] European Medicines Agency (2014). Community herbal monograph on ESCOP Eleutherococcus senticosus (Rupr. et Maxim.) Maxim., radix. EMA/HMPC/680618/2013. London, United Kingdom: European Medicines Agency.

[B133] European Pharmacopoeia (2019). European pharmacopoeia 10th edition. European Directorate for the Quality of Medicines and HealthCare. Strasbourg, France: Council of Europe.

[B129] European Pharmacopoeia (2019). European pharmacopoeia 10th edition. Strasbourg: Council of Europe.

[B23] Facchinetti F. Neri I. Tarabusi M. (2002). *Eleutherococcus senticosus* reduces cardiovascular stress response in healthy subjects: a randomized, placebo-controlled trial. Stress Health 18, 11–17. 10.1002/smi.914

[B25] Fei X. J Zhu L. L. Xia L. M. Peng W. B. Wang Q. (2014). *Acanthopanax senticosus* attenuates inflammation in lipopolysaccharide-induced acute lung injury by inhibiting the NF-κB pathway. Genet. Mol. Res. 13, 10537–10544. 10.4238/2014.December.12.16 25511038

[B26] Feng S. l. Hu F. Zhao J. X. Liu X. Li Y. (2006). Determination of Eleutheroside E and Eleutheroside B in rat plasma and tissue by high-performance liquid chromatography using solid-phase extraction and photodiode array detection. Eur. J. Pharm. Biopharm. 62, 315–320. 10.1016/j.ejpb.2005.09.007 16318914

[B27] Frantz S. Tillmanns J. Kuhlencordt P. J. Schmidt I. Adamek A. Dienesch C. (2007). Tissue-specific effects of the nuclear factor kappaB subunit p50 on myocardial ischemia-reperfusion injury. Am. J. Pathol. 171, 507–512. 10.2353/ajpath.2007.061042 17556593 PMC1934536

[B28] Gabrielian E. S. Shukarian A. K. Goukasova G. I. Chandanian G. L. Panossian A. G. Wikman G. (2002). A double blind, placebo-controlled study of *Andrographis paniculata* fixed combination Kan Jang in the treatment of acute upper respiratory tract infections including sinusitis. Phytomedicine 9, 589–597. 10.1078/094471102321616391 12487322

[B29] Gaffney B. T. Hügel H. M. Rich P. A. (2001). The effects of *Eleutherococcus senticosus* and *Panax ginseng* on steroidal hormone indices of stress and lymphocyte subset numbers in endurance athletes. Life Sci. 70, 431–442. 10.1016/s0024-3205(01)01394-7 11798012

[B30] Gerontakos S. Taylor A. Avdeeva A. Y. Shikova V. A. Pozharitskaya O. N. Casteleijn D. (2021). Findings of Russian literature on the clinical application of *Eleutherococcus senticosus* (Rupr. and Maxim.): a narrative review. J. Ethnopharmacol. 278, 114274. 10.1016/j.jep.2021.114274 34087398

[B31] Guo S. Wei H. Li J. Fan R. Xu M. Chen X. (2019). Geographical distribution and environmental correlates of eleutherosides and isofraxidin in *Eleutherococcus senticosus* from natural populations in forests at Northeast China. Forests 10, 872. 10.3390/f10100872

[B32] Han J. Pang X. Liao B. Yao H. Song J. Chen S. (2016). An authenticity survey of herbal medicines from markets in China using DNA barcoding. Sci. Rep. 6, 18723. 10.1038/srep18723 26740340 PMC4703975

[B33] Harkey M. R. Henderson G. L. Gershwin M. E. Stern J. S. Hackman R. M. (2001). Variability in commercial ginseng products: an analysis of 25 preparations. Am. J. Clin. Nutr. 73, 1101–1106. 10.1093/ajcn/73.6.1101 11382666

[B34] Hartz A. J. Bentler S. Noyes R. Hoehns J. Logemann C. Sinift S. (2004). Randomized controlled trial of Siberian ginseng for chronic fatigue. Psychol. Med. 34, 51–61. 10.1017/s0033291703008791 14971626

[B35] He Y. Wang Y. Zhang X. Zheng Z. Liu S. Xing J. (2020). Chemical characterization of small-molecule inhibitors of monoamine oxidase B synthesized from the *Acanthopanax senticosus* root with affinity ultrafiltration mass spectrometry. Rapid. Commun. Mass Spectrom. 34, e8694. 10.1002/rcm.8694 31826305

[B36] Hebert P. D. Cywinska A. Ball S. L. deWaard J. R. (2003). Biological identifications through DNA barcodes. Proc. Biol. Sci. 270, 313–321. 10.1098/rspb.2002.2218 12614582 PMC1691236

[B37] Hikino H. Takahashi M. Otake K. Konno C. (1986). Isolation and hypoglycemic activity of eleutherans A, B, C, D, E, F, and G: glycans of *Eleutherococcus senticosus* roots. J. Nat.Prod. 49, 293–297. 10.1021/np50044a015 3734812

[B38] Huang L. Zhao H. Huang B. Zheng C. Peng W. Qin L. (2011a). *Acanthopanax senticosus*: review of botany, chemistry and pharmacology. Pharmazie 66, 83–97. 10.1691/ph.2011.0744 21434569

[B39] Huang L. Z. Huang B. K. Ye Q. Qin L. P. (2011b). Bioactivity-guided fractionation for anti-fatigue property of *Acanthopanax senticosus* . J. Ethnopharmacol. 133, 213–219. 10.1016/j.jep.2010.09.032 20920564

[B41] Huang D. Hu Z. Yu Z. (2013). Eleutheroside B or E enhances learning and memory in experimentally aged rats. Neural. Regen. Res. 8, 1103–1112. 10.3969/j.issn.1673-5374.2013.12.005 25206404 PMC4145894

[B42] Huang Y. H. Li J. T. Zan K. Wang J. Fu Q. (2021). The traditional uses, secondary metabolites, and pharmacology of *Eleutherococcus* species. Phytochem. Rev. 21, 1081–1184. 10.1007/s11101-021-09775-z

[B17] Japanese Pharmacopoeia (2019). Japanese Pharmacopoeia 18th Edition. Tokyo, Japan: Ministry of Health, Labour and Welfare.

[B125] Japanese Pharmacopoeia (2021). Japanese pharmacopoeia 18th edition. Tokyo: Ministry of Health, Labour and Welfare.

[B43] Jia A. Zhang Y. Gao H. Zhang Z. Zhang Y. Wang Z. (2021). A review of *Acanthopanax senticosus* (Rupr and Maxim.) harms: from ethnopharmacological use to modern application. J. Ethnopharmacol. 268, 113586. 10.1016/j.jep.2020.113586 33212178

[B44] Jiang W. Li W. Han L. Liu L. Zhang Q. Zhang S. (2006). Biologically active triterpenoid saponins from *Acanthopanax senticosus* . J. Nat. Prod. 69, 1577–1581. 10.1021/np060195+ 17125224

[B45] Jin J. L. Lee S. Lee Y. Y. Kim J. M. Heo J. E. Yun-Choi H. S. (2004). Platelet anti-aggregating triterpenoids from the leaves of *Acanthopanax senticosus* and the fruits of A. sessiliflorus. Planta Med. 70, 564–566. 10.1055/s-2004-827159 15241893

[B46] Jin L. Schmiech M. El Gaafary M. Zhang X. Syrovets T. Simmet T. (2020). A comparative study on root and bark extracts of *Eleutherococcus senticosus* and their effects on human macrophages. Phytomedicine 68, 153181. 10.1016/j.phymed.2020.153181 32065954

[B47] Jung H. J Park H. J. Kim R. G. Shin K. M. Ha J. Choi J. W. (2003). *In vivo* anti-inflammatory and antinociceptive effects of liriodendrin isolated from the stem bark of *Acanthopanax senticosus* . Planta Med. 69, 610–616. 10.1055/s-2003-41127 12898415

[B48] Jung C. H. Jung H. Shin Y. C. Park J. H. Jun C. Y. Kim H. M. (2007). *Eleutherococcus senticosus* extract attenuates LPS-induced iNOS expression through the inhibition of Akt and JNK pathways in murine macrophage. J. Ethnopharmacol. 113, 183–187. 10.1016/j.jep.2007.05.023 17644291

[B49] Kil Y. S. Park J. Y. Kim Y. Nam S. J. Kim S. J. Kim Y. S. (2015). Utilization of circular dichroism experiment to distinguish acanthoside D and eleutheroside E. Arch. Pharm. Res. 38, 1921–1925. 10.1007/s12272-015-0586-7 25802110

[B50] Kim Y. H. Co M. L. Kim D. B. Shin G. H. Lee J. H. Lee J. S. (2015). The antioxidant activity and their major antioxidant compounds from *Acanthopanax senticosus* and *A. koreanum* . Molecules 20, 13281–13295. 10.3390/molecules200713281 26205054 PMC6331968

[B51] Koren G. Randor S. Martin S. Danneman D. (1990). Maternal ginseng use associated with neonatal androgenization. JAMA 264, 2866. 10.1001/jama.1990.03450220028007 2232076

[B52] Kurkin V. A. Zapesochnaya G. G. Bandyshev V. V. (1991). Phenolic compounds of *Eleutherococcus senticosus* . Chem. Nat. Compd. 27, 755–756. 10.1007/bf00629948

[B53] Lazarev N. V. (1958). General and specific in action of pharmacological agents. Farmakol. Toksikol. 21, 81–86. 13562193

[B54] Lee D. Park J. Yoon J. Kim M. Y. Choi H. Y. Kim H. (2012). Neuroprotective effects of *Eleutherococcus senticosus* bark on transient global cerebral ischemia in rats. J. Ethnopharmacol. 139, 6–11. 10.1016/j.jep.2011.05.024 21645606

[B55] Lee J. Jung E. Kim Y. S. Park D. Toyama K. Date A. (2013). Phloridzin isolated from *Acanthopanax senticosus* promotes proliferation of α6 integrin (CD 49f) and β1 integrin (CD29) enriched for a primary keratinocyte population through the ERK-mediated mTOR pathway. Arch. Dermatol. Res. 305, 747–754. 10.1007/s00403-013-1398-6 23912479

[B56] Li X. C. Barnes D. Khan I. (2001). A new lignan glycoside from *Eleutherococcus senticosus* . Planta Med. 67, 776–778. 10.1055/s-2001-18352 11731930

[B57] Li F. Li W. Fu H. Zhang Q. Koike K. (2007). Pancreatic lipase-inhibiting triterpenoid saponins from fruits of *Acanthopanax senticosus* . Chem. Pharm. Bull. 55, 1087–1089. 10.1248/cpb.55.1087 17603209

[B58] Li W. W. Guo H. Wang X. M. (2013). Relationship between endogenous hydrogen sulfide and blood stasis syndrome based on the Qi-blood theory of Chinese medicine. Chin. J. Integr. Med. 19, 701–705. 10.1007/s11655-013-1567-7 23975135

[B59] Li T. Ferns K. Yan Z. Q. Yin S. Y. Kou J. J. Li D. (2016). *Acanthopanax senticosus*: photochemistry and anticancer potential. Am. J. Chin. Med. 44, 1543–1558. 10.1142/S0192415X16500865 27852123

[B60] Li X. Chen C. Leng A. Qu J. (2021). Advances in the extraction, purification, structural characteristics and biological activities of *Eleutherococcus senticosus* polysaccharides: a promising medicinal and edible resource with development value. Front. Pharmacol. 12, 753007. 10.3389/fphar.2021.753007 34790125 PMC8591254

[B61] Lim S. S. Lee J. M. Park H. S. Cho S. H. Shin K. H. Lee S. H. (2007). GC/MS analysis of volatile constituents from *Acanthopanax senticosus* . Kor. J. Pharmacogn. 38, 327–333.

[B62] Lin Q. Y. Jin L. J. Ma Y. S. Shi M. Xu J. P. (2007). *Acanthopanax senticosus* inhibits nitric oxide production in murine macrophages *in vitro* and *in vivo* . Phytother. Res. 21, 879–883. 10.1002/ptr.2171 17514632

[B63] Lin Q. Y. Jin L. J. Cao Z. H. Xu J. P. (2008). Inhibition of inducible nitric oxide synthase by *Acanthopanax senticosus* extract in RAW264.7 macrophages. J. Ethnopharmacol. 118, 231–236. 10.1016/j.jep.2008.04.003 18486372

[B64] Liu S. M. Li X. Z. Zhang S. N. Yang Z. M. Wang K. X. Lu F. (2018). *Acanthopanax senticosus* protects structure and function of mesencephalic mitochondria in A mouse model of parkinson’s disease. Chin. J. Integr. Med. 24, 835–843. 10.1007/s11655-018-2935-5 30090975

[B65] Liu Y. Jiang P. Zhang M. L. Pan J. Guan W. Li X. M. (2020). Triterpenoid saponins from the fruit of *Acanthopanax senticosus* (Rupr. and Maxim.) harms. Front. Chem. 10, 825763. 10.3389/fchem.2022.825763 35265584 PMC8899614

[B66] Majnooni M. B. Fakhri S. Shokoohinia Y. Mojarrab M. Kazemi-Afrakoti S. Farzae i M. H. (2020). Isofraxidin: synthesis, biosynthesis, isolation, pharmacokinetic and pharmacological properties. Molecules 25, 2040. 10.3390/molecules25092040 32349420 PMC7248759

[B67] Makarieva T. Dmitrenok A. S. Stonik V. Patel A. V. Canfield L. M. (1997). Lignans from *Eleutherococcus senticosus* (Siberian ginseng). Pharm. Sci. 3, 525–527. 10.1111/j.2042-7158.1997.tb00487.x

[B68] Maruyama T. Kamakura H. Miyai M. Komatsu K. Kawasaki T. Fujita M. (2008). Authentication of the traditional medicinal plant *Eleutherococcus senticosus* by DNA and chemical analyses. Planta Med. 74, 787–789. 10.1055/s-2008-1074537 18500683

[B69] McNaughton L. Egan G. Caelli G. A. (1989). Comparison of Chinese and *Russian ginseng* as ergogenic aids to improve various facets of physical fitness. Int. J.Clin. Nutr. Rev. 9, 32–35.

[B70] Melchior J. Spasov A. A. Ostrovskij O. V. Bulanov A. E. Wikman G. (2000). Double-blind, placebo-controlled pilot and phase III study of activity of standardized *Andrographis paniculata* herba nees extract fixed combination (*Kan jang*) in the treatment of uncomplicated upper-respiratory tract infection. Phytomedicine 7, 341–350. 10.1016/S0944-7113(00)80053-7 11081985

[B71] Miyazaki S. Oikawa H. Takekoshi H. Hoshizaki M. Ogata M. Fujikawa T. (2019). Anxiolytic effects of *Acanthopanax senticosus* HARMS occur *via* regulation of autonomic function and activate hippocampal BDNF–TrkB signaling. Molecules 24, 132. 10.3390/molecules24010132 30602695 PMC6337493

[B72] Murray M. T. (2020). “76 - *eleutherococcus senticosus* (Siberian ginseng),” in Textbook of natural medicine. Fifth Edition (Churchill Livingstone). 10.1016/B978-0-323-43044-9.00076-5

[B73] Narimanian M. Badalyan M. Panosyan V. Gabrielyan E. Panossian A. Wikman G. (2005a). Randomized trial of a fixed combination (KanJang) of herbal extracts containing *Adhatoda vasica*, *Echinacea purpurea* and *Eleutherococcus senticosus* in patients with upper respiratory tract infections. Phytomedicine 12, 539–547. 10.1016/j.phymed.2004.10.001 16121513

[B74] Narimanian M. Badalyan M. Panosyan V. Gabrielyan E. Panossian A. Wikman G. (2005b). Impact of Chisan (ADAPT-232) on the quality-of-life and its efficacy as an adjuvant in the treatment of acute non-specific pneumonia. Phytomedicine 12, 723–729. 10.1016/j.phymed.2004.11.004 16323290

[B75] Ngwa C. Liu F. (2019). CD200-CD200R signaling and diseases: a potential therapeutic target? Int. J. Physiol. Pathophysiol. Pharmacol. 11, 297–309. 31993106 PMC6971504

[B76] Online TWF. Available online at: http://www.worldfloraonline.org/taxon/wfo-4000013194#children (Accessed on April 06, 2024).

[B77] Panossian A. (2017). Understanding adaptogenic activity: specificity of the pharmacological action of adaptogens and other phytochemicals. Ann. N. Y. Acad. Sci. 1401, 49–64. 10.1111/nyas.13399 28640972

[B78] Panossian A. Wagner H. (2005). Stimulating effect of adaptogens: an overview with particular reference to their efficacy following single dose administration. Phytother. Res. 19, 819–838. 10.1002/ptr.1751 16261511

[B79] Panossian A. Wikman G. (2009). Evidence-based efficacy of adaptogens in fatigue, and molecular mechanisms related to their stress-protective activity. Curr. Clin. Pharmacol. 4, 198–219. 10.2174/157488409789375311 19500070

[B80] Panossian A. G. Efferth T. Shikov A. N. Pozharitskaya O. N. Kuchta K. Mukherjee P. K. (2021). Evolution of the adaptogenic concept from traditional use to medical systems: pharmacology of stress‐ and aging‐related diseases. Med. Res. Rev. 41, 630–703. 10.1002/med.21743 33103257 PMC7756641

[B81] Park H. R. Park E. Rim A. R. Jeon K. I. Hwang J. H. Lee S. C. (2006). Antioxidant activity of extracts from *Acanthopanax senticosus* . Afr. J. Biotechnol. 5, 2388–2396. 10.5897/AJB06.606

[B82] Price R. B. Duman R. (2020). Neuroplasticity in cognitive and psychological mechanisms of depression: an integrative model. Mol. Psychiatry. 25, 530–543. 10.1038/s41380-019-0615-x 31801966 PMC7047599

[B83] Richter R. Hanssen H. P. Koenig W. A. Koch A. (2007). Essential Oil Composition of *Eleutherococcus senticosus* (Rupr. et Maxim.) Maxim Roots. J. Essen. Oil Res. 19, 209–210. 10.1080/10412905.2007.9699262

[B84] Ritzel R. M. Al Mamun A. Crapser J. Verma R. Patel A. R. Knight B. E. (2019). CD200-CD200R1 inhibitory signaling prevents spontaneous bacterial infection and promotes resolution of neuroinflammation and recovery after stroke. J. Neuroinflammation 16, 40. 10.1186/s12974-019-1426-3 30777093 PMC6378746

[B85] Ruhsam M. Hollingsworth P. (2017). Authentication of Eleutherococcus and Rhodiola herbal supplement products in the United Kingdom. J. Pharm. Biomed. Anal. 149, 403–409. 10.1016/j.jpba.2017.11.025 29154110

[B86] Schaffler K. Wolf O. T. Burkart M. (2013). No benefit adding *eleutherococcus senticosus* to stress management training in stress-related fatigue/weakness, impaired work or concentration, a randomized controlled study. Pharmacopsychiatry 46, 181–190. 10.1055/s-0033-1347178 23740477

[B87] Schmolz M. W. Sacher F. Aicher B. (2001). The synthesis of Rantes, G-CSF, IL-4, IL-5, IL-6, IL-12 and IL-13 in human whole-blood cultures is modulated by an extract from *Eleutherococcus senticosus* L. roots. Phytother. Res. 15, 268–270. 10.1002/ptr.746 11351368

[B88] Segiet-Kujawa E. Kaloga M. (1991). Triterpenoid saponins of *Eleutherococcus senticosus* roots. J. Nat.Prod. 1 (54), 1044–1048. 10.1021/np50076a018

[B89] Shi X. Yang Y. Ren H. Sun S. Mu L. T. Chen X. (2020). Identification of multiple components in deep eutectic solvent extract of *Acanthopanax senticosus* root by ultra-high-performance liquid chromatography with quadrupole orbitrap mass spectrometry. Phytochem. Lett. 35, 175–185. 10.1016/j.phytol.2019.11.017

[B90] Shikov A. N. Narkevich I. A. Flisyuk E. V. Luzhanin V. G. Pozharitskaya O. N. (2021). Medicinal plants from the 14th edition of the Russian Pharmacopoeia, recent updates. J. Ethnopharmacol. 268, 113685. 10.1016/j.jep.2020.113685 33309919

[B91] Slacanin I. Marston A. Hostettmann K. Guédon D. Abbe P. (1991). The isolation of *Eleutherococcus senticosus* constituents by centrifugal partition chromatography and their quantitative determination by high performance liquid chromatography. Phytochem. Anal. 2, 137–142. 10.1002/pca.2800020310

[B92] Smalinskiene A. Lesauskaite V. Zitkevicius V. Savickas A. Ryselis S. Sadauskiene I. (2009). Estimation of the combined effect of *Eleutherococcus senticosus* extract and cadmium on liver cells. Ann. N. Y. Acad. Sci. 1171, 314–320. 10.1111/j.1749-6632.2009.04678.x 19723071

[B93] Song C. Li S. Duan F. Liu M. Shan S. Ju J. (2022). The therapeutic effect of *Acanthopanax senticosus* components on radiation-induced brain injury based on the pharmacokinetics and neurotransmitters. Molecules 27, 1106. 10.3390/molecules27031106 35164373 PMC8839712

[B126] State Pharmacopoeia of Russian Federation. (2018). The State Pharmacopoeia of Russian Federation 14th edition. Moscow, Russia: Ministry of Public Health of the Russian Federation.

[B94] Su X. Liu B. Gong F. Yin J. Sun Q. Gao Y. (2019). Isofraxidin attenuates IL-1β-induced inflammatory response in human nucleus pulposus cells. J. Cell. Biochem. 120, 13302–13309. 10.1002/jcb.28604 30891836

[B95] Su J. Wang Q. Li Z. Feng Y. Li Y. Yang S. (2021). Different metabolites in the roots, seeds, and leaves of *Acanthopanax senticosus* and their role in alleviating oxidative stress. J. Anal. Methods Chem. 2021, 6628880. 10.1155/2021/6628880 33954008 PMC8064801

[B96] Su J. Zhang X. Kan Q. Chu X. (2022). Antioxidant activity of *Acanthopanax senticosus* flavonoids in H_2_O_2_-induced RAW 264.7 cells and DSS-induced colitis in mice. Molecules 27, 2872. 10.3390/molecules27092872 35566218 PMC9101407

[B97] Szołomicki J. Samochowiec L. Wójcicki J. Droździk M. Szołomicki S. (2000). The influence of active components of *Eleutherococcus senticosus* on cellular defence and physical fitness in man. Phytother. Res. 14, 30–35. 10.1002/(sici)1099-1573(200002)14:1<30::aid-ptr543>3.3.co;2-m 10641044

[B98] Tan J. Luo J. Meng C. Jiang N. Cao J. Zhao J. (2021). Syringin exerts neuroprotective effects in a rat model of cerebral ischemia through the FOXO3a/NF-κB pathway. Int. Immunopharmacol. 90, 107268. 10.1016/j.intimp.2020.107268 33316740

[B99] Thom E. Wollan T. (1997). A controlled clinical Study of Kanjang mixture in the treatment of uncomplicated upper respiratory tract infections. Phytother. Res. 11, 207–210. 10.1002/(sici)1099-1573(199705)11:3<207::aid-ptr82>3.0.co;2-s

[B100] Tohda C. Ichimura M. Bai Y. Tanaka K. Zhu S. Komatsu K. (2008). Inhibitory effects of *Eleutherococcus senticosus* extracts on amyloid beta(25-35)-induced neuritic atrophy and synaptic loss. J. Pharmacol. Sci. 107, 329–339. 10.1254/jphs.08046fp 18612196

[B101] Tolonen A. Joutsamo T. Mattlla S. Kämäräinen T. Jalonen J. (2002). Identification of isomeric dicaffeoylquinic acids from *Eleutherococcus senticosus* using HPLC-ESI/TOF/MS and 1H-NMR methods. Phytochem. Anal. 13, 316–328. 10.1002/pca.663 12494749

[B103] Wang S. Yang X. (2020). Eleutheroside E decreases oxidative stress and NF-κB activation and reprograms the metabolic response against hypoxia-reoxygenation injury in H9c2 cells. Int. Immunopharmacol. 84, 106513. 10.1016/j.intimp.2020.106513 32330867

[B104] Wang D. Liu F. Zhu L. Lin P. Han F. Wang X. (2020). FGF21 alleviates neuroinflammation following ischemic stroke by modulating the temporal and spatial dynamics of microglia/macrophages. J. Neuroinflammation 17, 257. 10.1186/s12974-020-01921-2 32867781 PMC7457364

[B105] WHO (2002). WHO monographs on selected medicinal plants, volume 2. Geneva: WHO, 83–96.

[B106] Wu Z. Li W. Sun Y. Fu K. Cheng S. (2017). Eleutheroside E inhibits doxorubicin-induced inflammation and apoptosis in rat cardiomyocytes by modulating activation of NF-κB pathway. Trop. J. Pharm. Res. 16, 515–523. 10.4314/tjpr.v16i3.4

[B107] Xia Y. G Gong F. Q. Guo X. D. Song Y. Li C. X. Liang J. (2019). Rapid screening and characterization of triterpene saponins in *Acanthopanax senticosus* leaves *via* untargeted MS(All) and SWATH techniques on a quadrupole time of flight mass spectrometry. J. Pharm. Biomed. Anal. 170, 68–82. 10.1016/j.jpba.2019.02.032 30909056

[B108] Xie Y. Zhang B. Zhang Y. (2015). Protective effects of Acanthopanax polysaccharides on cerebral ischemia-reperfusion injury and its mechanisms. Int. J. Biol. Macromol. 72, 946–950. 10.1016/j.ijbiomac.2014.09.055 25451748

[B109] Yamazaki T. Shimosaka S. Sakurai M. Matsumura T. Tsukiyama T. Tokiwa T. (2004). Anti-inflammatory effects of a major component of *Acanthopanax senticosus* Harms, isofraxidin. Seibutsu Butsuri Kagaku 48, 55–58. 10.2198/sbk.48.55

[B110] Yamazaki T. Matsumura T. Tsukiyama T. Tokiwa T. (2006). Anti-inflammatory effects of eleutheroside E from *Acanthopanax senticosus* . Tissue Cult. Res. Commun. 25, 137–145. 10.11418/jtca1981.25.3-4_137

[B112] Yan Z. Liu J. Lu D. Narlawar R. Groundwater P. Li P. (2014). Two new ceramides from the fruit pulp of *Acanthopanax senticosus* (Rupr. Et Maxim) Harms. Nat. Prod. Res. 28, 144–149. 10.1080/14786419.2013.856908 24261557

[B113] Yan-Lin S. Lin-De L. Soon-Kwan H. (2011). *Eleutherococcus senticosus* as a crude medicine: review of biological and pharmacological effects. J. Med. Plants Res. 5, 5946–5952. 10.5897/JMPR11.728

[B114] Yang R. Meng X. Zhao W. Xu S. Q. Wang S. Y. Li M. M. (2025). Phenylpropanoids of *Eleutherococcus senticosus* (Rupr. and Maxim.) maxim. Alleviate oxidative stress in Alzheimer’s disease *in vitro* and *in vivo* models by regulating Mst1 and affecting the Nrf2/Sirt3 pathway. Bioorg. Chem. 159, 108347. 10.1016/j.bioorg.2025.108347 40081261

[B115] Yat P. N. Arnason J. T. Awang D. V. C. (1998). An improved extraction procedure for the rapid, quantitative high performance liquid chromatographic estimation of the main eleutherosides (B and E) in *Eleutherococcus senticosus* (eleuthero). Phytochem. Anal. 9, 291–295. 10.1002/(sici)1099-1565(199811/12)9:6<291::aid-pca417>3.0.co;2-k

[B116] Yu W. Zhang H. Huang W. Chen J. Liang X. (2006). Analysis of the volatile oil from the stem of *Acanthopanax senticosus* (Rupr. et Maxim.) harms with several hyphenated methods of chromatography. Front. Chem. China 1, 193–198. 10.1007/s1458-006-0008-y 16013569

[B117] Załuski D. Kuźniewski R. (2016). *In vitro* Anti-AChE, Anti-BuChE, and antioxidant activity of 12 extracts of eleutherococcus species. Oxid. Med. Cell. Longev. 2016, 4135135. 10.1155/2016/4135135 27803761 PMC5075622

[B118] Załuski D. Smolarz H. (2015). Influence of Polish climate conditions on content and the chemical variation of volatiles in the roots of six eleutherococcus species and their potential use. Rec. Nat. Prod. 10, 240–244.

[B119] Załuski D. Kuźniewski R. Janeczko Z. (2018). HPTLC-profiling of eleutherosides, mechanism of antioxidative action of eleutheroside E1, the PAMPA test with LC/MS detection and the structure–activity relationship. Saudi J. Biol. Sci. 25, 520–528. 10.1016/j.sjbs.2016.01.018 29692653 PMC5911645

[B120] Zgórka G. Kawka S. (2001). Application of conventional UV, photodiode array (PDA) and fluorescence (FL) detection to analysis of phenolic acids in plant material and pharmaceutical preparations. J. Pharm. Biomed. Anal. 24, 1065–1072. 10.1016/s0731-7085(00)00541-0 11248502

[B121] Zhang H. Gu H. Jia Q. Zhao Y. Li H. Shen S. (2020). Syringin protects against colitis by ameliorating inflammation. Arch. Biochem. Biophys. 680, 108242. 10.1016/j.abb.2019.108242 31899146

[B122] Zhang Z. Wu Y. Shi D. Jiang C. Cao H. Jiang F. (2024). *Acanthopanax senticosus* improves cognitive impairment in Alzheimer’s disease by promoting the phosphorylation of the MAPK signaling pathway. Front. Immunol. 15, 1383464. 10.3389/fimmu.2024.1383464 38545117 PMC10965608

[B128] Zhu S. Bai Y. Oya M. Tanaka K. Komatsu K. Maruyama T. (2011). Genetic and chemical diversity of *Eleutherococcus senticosus* and molecular identification of Siberian ginseng by PCR-RFLP analysis based on chloroplast trnK intron sequence. Food Chem. 129, 1844–1850. 10.1016/j.foodchem.2011.05.128

